# Multiple Reaction Monitoring for quantitative laccase kinetics by LC-MS

**DOI:** 10.1038/s41598-018-26523-0

**Published:** 2018-05-25

**Authors:** Valentina Perna, Jane W. Agger, Jesper Holck, Anne S. Meyer

**Affiliations:** Center for BioProcess Engineering, Department of Chemical and Biochemical Engineering, Technical University of Denmark, Kgs, Lyngby, 2800 Denmark

## Abstract

Laccases (EC 1.10.3.2) are enzymes known for their ability to catalyse the oxidation of phenolic compounds using molecular oxygen as the final electron acceptor. Lignin is a natural phenylpropanoids biopolymer whose degradation in nature is thought to be aided by enzymatic oxidation by laccases. Laccase activity is often measured spectrophotometrically on compounds such as syringaldazine and ABTS which poorly relate to lignin. We employed natural phenolic hydroxycinnamates having different degree of methoxylations, *p*-coumaric, ferulic and sinapic acid, and a lignin model OH-dilignol compound as substrates to assess enzyme kinetics by HPLC-MS on two fungal laccases *Trametes versicolor* laccase, Tv and *Ganoderma lucidum* laccase, Gl. The method allowed accurate kinetic measurements and detailed insight into the product profiles of both laccases. Both Tv and Gl laccase are active on the hydroxycinnammates and show a preference for substrate with methoxylations. Product profiles were dominated by the presence of dimeric and trimeric species already after 10 minutes of reaction and similar profiles were obtained with the two laccases. This new HPLC-MS method is highly suitable and accurate as a new method for assaying laccase activity on genuine phenolic substrates, as well as a tool for examining laccase oxidation product profiles.

## Introduction

Lignin is a natural plant biopolymer composed of aromatic units in the form of phenylpropanoids, i.e. p-hydroxyphenyl (H), guaiacyl (G), and syringyl (S)^1^. Lignin is present at levels of 20–50% by weight in lignocellulosic materials. Along with cellulosic biomass refining, the attention on exploiting lignin for sustainable production of new materials or for recovering valuable aromatic compounds has recently risen significantly^2^. Degradation of lignin in nature is thought to be achieved by means of enzymes such as peroxidases and laccases produced by different organisms. Laccases (benzenediol: oxygen oxidoreductases; EC 1.10.3.2) are blue multi-copper oxidoreductase enzymes produced ubiquitously by fungi, plants and bacteria (and even humans)^3,4^ currently receiving particular attention because they catalyse the oxidation of phenolic compounds similar to subunits in lignin using only molecular oxygen as final electron acceptor^5^. The overall reaction cycle involves the oxidation of four moles of phenolic substrate with the simultaneous reduction of one mole of O_2_ to two moles of H_2_O^6,7^. As a result of the enzyme catalysed phenol oxidation, phenoxy radicals are generated in lignin. In turn, the phenoxy radicals react further via different non-enzymatic reactions such as radical polymerization, depolymerization, grafting and modification of functional groups^5,8–10^.

Often laccase activity is determined by monitoring the oxidation of chemical compounds such as syringaldazine (4-hydroxy-3,5-dimethoxy-benzaldehyde azine) and ABTS (2,2′-azino-bis(3-ethylbenzothiazoline-6-sulphonic acid)) by changes in UV absorbance. Since syringaldazine and ABTS are poorly related to lignin, the use of them provide insufficient information about the actual oxidative kinetics of laccase on true natural phenols and “lignin-like” phenolic structures. Another drawback of using these substrates is that the oxidation products formed after laccase oxidation tend to precipitate quickly even at modest concentrations^11^ creating an unstable assay with the risk of measuring wrong enzyme activity values. Pardo *et al*.^12,13^ developed a new colorimetric assay method using “lignin-like” compounds including sinapic acid and 2,6-dimethoxyphenol, where the oxidation was followed spectrophotometrically. The substrates chosen are highly relevant but spectrophotometric analysis only allows sensitivity in the nmol range^12^ and provides little insight into the possible products formed during the laccase catalysed oxidation.

HPLC-MS analysis allows for direct monitoring of product formation and quantitative assessment of kinetics. The direct monitoring of the reaction in real time not only give information about the substrate oxidation but also provide insight into the products formed during the continued radical reactions, including e.g. various isomeric and polymeric compounds. HPLC-MS is receiving increasing attention as a quantitative tool and particularly within the field of proteomics the quantitative use of Multiple Reaction Monitoring is widespread^14^. Multiple Reaction Monitoring is based on identification and quantification of specific fragment ions from a predetermined precursor list of parent masses and offers several advantages including high level of specificity, high sensitivity and low susceptibility to interfering compounds^15,16^. The latter is particularly important in biological samples with highly complex matrices.

The objective of the present work was to develop a real time, highly sensitive and accurate methodology based on HPLC-MS to assess laccase activity on monomeric phenolics and a dimeric OH-lignol-compound and specifically assess whether the kinetic rates and catalytic efficiencies of laccases depend on the substitutions on the phenolic ring structure and/or on the molecular structure as a whole beyond the monomeric phenol moiety. The work performed was driven by the hypothesis that laccases may have different reaction kinetics on different hydroxycinnamates and that accurate and sensitive analysis might reveal such differences. We hypothesized that a methodology based on HPLC-MS analysis can be used to describe laccase oxidized reactions to a higher detail than what has been common practice both with respect to quantitative measures and product description. In this scenario Multiple Reaction Monitoring quantification by HPLC-MS is feasible because the reactions are well-defined consisting of known starting components.

**Figure 1:**
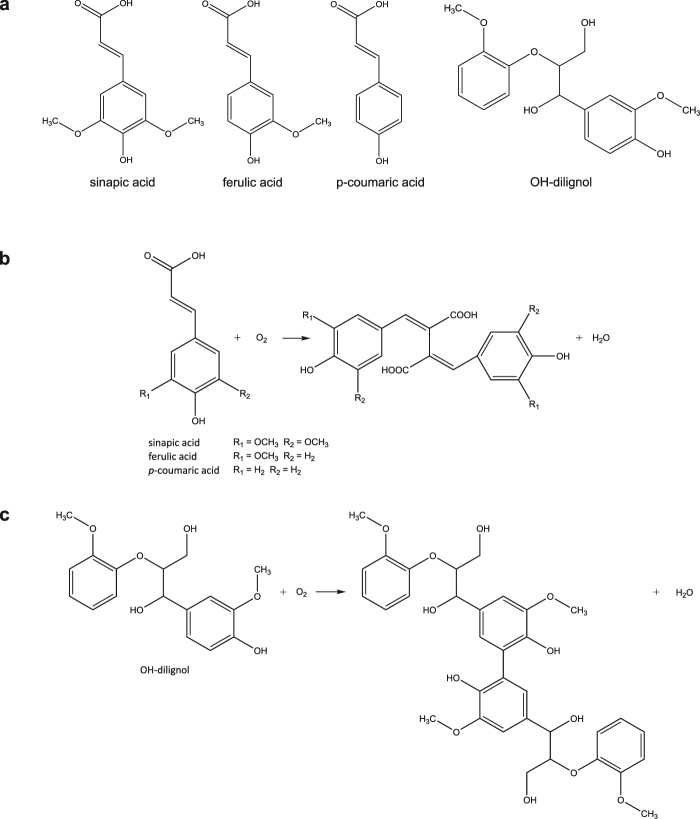
Hydroxycinnamic acid and OH-dilignol structures (**a**). Possible concept for product formation after laccase oxidation: dimer formation from the hydroxycinnamic acids (**b**) and from the OH-dilignol (**c**).

Three different hydroxycinnamic acids (sinapic acid, ferulic acid and *p*-coumaric acid) and an OH-dilignol (Fig. 1 panel a) were used to assess the action of two different white-rot fungal laccases: laccase from *Trametes versicolor* (Tv) and laccase from *Ganoderma lucidum* (Gl). The Tv laccase is a widely studied high redox potential laccase, whereas the Gl laccase represents a newer laccase, we have found to work particularly well in relation to enhancing cellulose catalysed lignocellulosic degradation^17^. From the type of the reactions monitored in this study, it is expected that oxidation products will primarily be dimeric and possibly similar to the structures in Fig. 1 panel b and c.

## Results

### Kinetics

**Figure 2:**
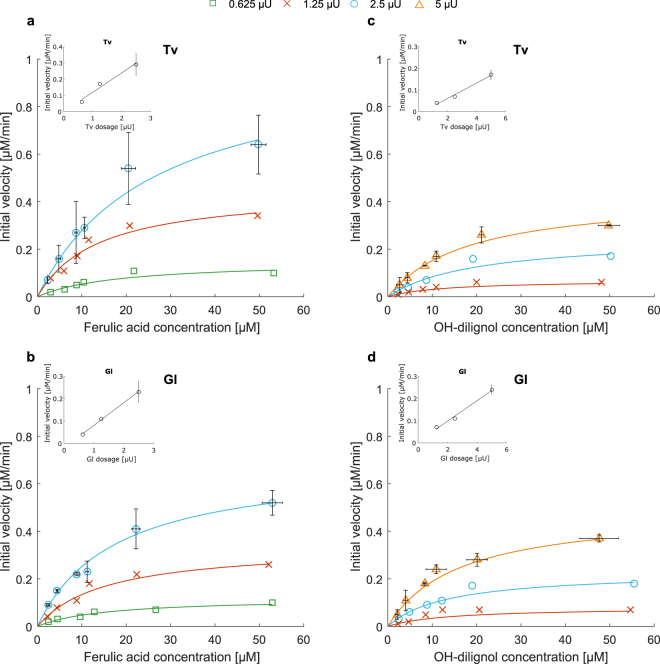
Michaelis-Menten curves for Tv and Gl laccase on ferulic acid (**a**,**b**) and OH-dilignol (**c**,**d**). Three different enzyme dosages (in syringaldazine assay units) are shown: for ferulic acid (**a**,**b**): 0.625 *μ*U (green open square), 1.25 *μ*U (red cross) and 2.5 *μ*U (blue open circle). For OH-dilignol (**c**,**d**): 1.25 *μ*U (red cross), 2.5 *μ*U (blue open circle) and 5 *μ*U (orange open triangle). Dose response at 10 *μ*M substrate concentration is shown in the inset. For the highest enzyme dose standard deviations are shown.

LC-MS was used to determined kinetics for *Trametes versicolor* (Tv) and *Ganoderma lucidum* (Gl) laccase on lignin model compounds: sinapic acid, ferulic acid, *p*-coumaric acid and OH-dilignol. Kinetics were determined as an on-line measurement of substrate depletion and these results moreover confirm that LC-MS analysis is a highly relevant methodology for describing enzyme kinetics. In order to overcome differences in enzyme purity between the Tv and Gl laccase the enzyme loading in the kinetic measurements was based on dosing equal levels of each enzyme’s activity on syringaldazine. In this way the dosage of active enzyme protein was uniform in all experiments allowing for direct comparison between the kinetic rates of the enzymes on the particular phenolic substrates. This approach for dosing is commonly used in enzyme work^12,18–21^ and represents another strategy for standardizing enzyme loading within a set of experiments in comparison to loading by protein concentration. As exemplified by ferulic acid and OH-dilignol (Fig. 2) there was a clear dose response effect for both enzymes where the initial rate increased perfectly proportionally with the increase in enzyme concentration on both substrates (Fig. 2 inserts). Equivalent results were obtained with both enzymes for the enzyme kinetics on sinapic acid and *p*-coumaric acid (Supplementary Fig. S1).

For both enzymes the K_m_ on the *p*-coumaric acid was significantly higher than the one for ferulic and sinapic acid, although no statistical significance could be discerned, the K_m_ values tended to decrease with the methoxylation (Table 1). The order of K_m_ on the hydroxycinnamates is in complete agreement with previous studies^19,22,23^. V_max_ is enzyme dosage dependent and should therefore only be compared between enzymes and both Tv and Gl showed the same overall maximum velocity on all the substrates except for ferulic acid where Tv laccase was slightly faster. Apparent specific activity is standardized with the enzyme dosage and allows comparison between substrates, confirming that the degree of methoxylation is affecting the enzyme activity (Table 2). The apparent catalytic efficiency is the number of oxidation cycles that the enzyme is capable of. The catalytic efficiency also demonstrates the influence of methoxylations where ferulic acid and sinapic acid show 2 to 3 order of magnitude higher apparent catalytic efficiency values than *p*-coumaric acid (Table 2). The poor catalytic efficiency obtained with both enzymes on *p*-coumaric acid is also a direct consequence of the high K_m_ value towards this substrate (Tables 1 and 2).

**Table 1:** K_m_ and V_max_ for hydroxycinnamic acids and OH-dilignol.

		Tv	Gl
Sinapic acid	V_max_ [*μ*M/min]	1.15 ± 0.07^x^	1.06 ± 0.03^x^
	K_m_ [*μ*M]	12.13 ± 2.23^b,x^	12.11 ± 0.46^b,x^
Ferulic acid	V_max_ [*μ*M/min]	1.14 ± 0.10^x^	0.82 ± 0.07^y^
	K_m_ [*μ*M]	17.17 ± 4.99^b,x^	17.84 ± 0.93^b,x^
*p*-coumaric acid	V_max_ [*μ*M/min]	6.73 ± 0.40^x^	7.31 ± 0.55^x^
	K_m_ [*μ*M]	130.27 ± 2.56^a,x^	271.04 ± 5.25^a,y^
OH-dilignol	V_max_ [*μ*M/min]	0.39 ± 0.03^x^	0.46 ± 0.03^x^
	K_m_ [*μ*M]	12.89 ± 3.68^b,x^	11.76 ± 2.61^b,x^

The enzymes were dosed at equal levels of syringaldazine activity. Standard deviations are shown and significant difference (p ≤ 0.05) of K_m_ column-wise are shown as superscripted letters (a-b), significance difference (p ≤ 0.05) of K_m_ and V_max_ row-wise are shown as superscripted letters (x−y).

**Table 2:** Apparent specific activity and apparent catalytic efficiency for hydroxycinnamic acids and OH-dilignol.

		Tv	Gl
Sinapic acid	Apparent specific activity [nM/*μ*U ⋅ s]	8.32 ± 0.47^a,x^	6.97 ± 0.22^a,x^
	Apparent catalytic efficiency [1/*μ*U ⋅ s]	565.6 ± 102.15^a,x^	550.8 ± 36.3^a,x^
Ferulic acid	Apparent specific activity [nM/*μ*U ⋅ s]	6.66 ± 0.59^a,x^	4.55 ± 0.45^b,y^
	Apparent catalytic efficiency [1/*μ*U ⋅ s]	280.0 ± 64.2^b,x^	284.8 ± 33.7^b,x^
*p*-coumaric acid	Apparent specific activity [nM/*μ*U ⋅ s]	0.51 ± 0.03^b,x^	0.55 ± 0.04^d,x^
	Apparent catalytic efficiency [1/*μ*U ⋅ s]	3.92 ± 0.27^c,x^	2.05 ± 0.17^c,y^
OH-dilignol	Apparent specific activity [nM/*μ*U ⋅ s]	1.43 ± 0.09^c,x^	1.70 ± 0.09^c,x^
	Apparent catalytic efficiency [1/*μ*U ⋅ s]	84.9 ± 20.5^d,x^	106.1 ± 20.8^d,x^

Apparent specific activity is defined as the amount of substrate that is converted by the enzyme in one second, the apparent catalytic efficiency is the number of oxidation cycles that the enzyme is capable of in one second. The enzymes were dosed at equal levels of syringaldazine activity. Standard deviations are shown and significant difference (p ≤ 0.05) of apparent specific activity and apparent catalytic efficiency column-wise are shown as superscripted letters (a–d), significance difference (p ≤ 0.05) of apparent specific activity and apparent catalytic efficiency row-wise are shown as superscripted letters (x–y).

OH-dilignol was chosen based on its similar structure to subunits in lignin and the fact that it represents a dimer. The K_m_ value for both enzymes towards this substrate was the same as the K_m_ values for the methoxylated hydroxycinnamates sinapic and ferulic acid, confirming that the presence of methoxylation is apparently of higher importance for the enzyme affinity than the remaining molecular structure. Despite similar affinities between the methoxylated hydroxycinnamate and OH-dilignol the apparent specific activity was approximately 2 to 4 times lower for the OH-dilignol and this was also reflected in the apparent catalytic efficiency (Table 2).

### Reaction evolution profiles

**Figure 3:**
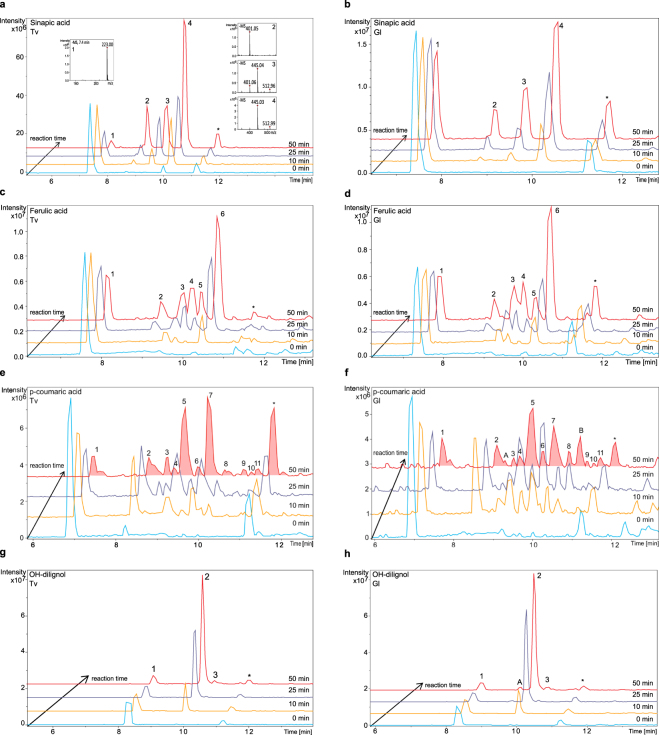
Laccase oxidation evolution profiles for hydroxycinnamic acids and OH-dilignol: oxidation of sinapic acid with Tv and Gl laccase (**a**,**b**); oxidation of ferulic acid with Tv and Gl laccase (**c**,**d**); oxidation of *p*-coumaric acid with Tv and Gl laccase (**e**,**f**) and oxidation of OH-dilignol with Tv and Gl laccase (**g**,**h**). Chromatograms at different reaction times are shown: 0 minutes (light blue), 10 minutes (yellow), 25 minutes (violet) and 50 minutes (red). Please note that the intensity scale may differ between chromatograms and is adjusted to give optimal display of figures. Panel a includes an example of the MS spectra corresponding to each peak, all ions are observed as [M − H]^−^. All other MS and MS/MS spectra are found in Supplementary Figs S2–S11. Oxidation reaction products are numbered according to substrate and are therefore comparable between enzymes.

The HPLC-MS methodology provides an insight into the products generated as a result of laccase driven substrate oxidation (Fig. 3). When looking into the product profiles generated during laccase oxidation of the hydroxycinnamates and OH-dilignol, it was observed that both enzymes generated similar products on each substrates (Fig. 3), although the oxidation of *p*-coumaric acid and OH-dilignol catalysed by the Gl laccase resulted in a few more peaks than those obtained with Tv laccase catalysis (Fig. 3 panel e, f, g and h).

The primary products detected were in agreement with the masses of dimers and trimers of the corresponding substrates but also compounds with loss of 44 were formed which could represent the loss of a carboxyl group. One example of the formation of such compounds is the reaction from sinapic acid (Fig. 3 panel a) where the MS spectra of products show two different isomers of dimer formation (peak 3 and 4) and two dimeric products presumably lacking a carboxylic group (peak 2 and 3). The work by Lackii *et al*.^24^ reports that the predominant coupling among sinapic acid radicals is the *β*−*β*′ coupling having a dehydrodisinapic lactone as main product. In accordance to that ferulic acid radicals may also have a *β*−*β*′ coupling obtaining a dehydrodiferulic acid lactone^25,26^. Suggested product structures can be found in Supplementary Figs S12–S15.

Reaction products are a result of (non-enzyme catalysed) chemical reactions between radicals formed by laccase oxidation and hence several conformations are possible. The data obtained show that such reactions may also lead to further random modification like decarboxylation. It appears that dimeric products occur early, and that decarboxylation may follow instantaneously (Fig. 3 panel a, b, c and d). At a later stage (after 25 minutes) in the reaction also trimeric and tetrameric products occurred (for *p*-coumaric acid in particular), some which might be further modified by decarboxylation. In the evolution of reaction products from *p*-coumaric acid oxidation certain products decrease in intensity after 50 minutes incubation (Fig. 3 panel e and f for peak number 2, 4, 6 and 8) and this may be a result of either compound instability or because the products react further with newly formed radicals from the laccase catalysis. The method does not allow the distinction between the two possibilities.

Laccase oxidation of OH-dilignol results in only two product peaks (Fig. 3 panel g and h), both dimeric after 50 minutes incubation and neither of these products are prone to decarboxylation. Furthermore, in order to fit the observed product masses, the OH-dilignol structure only allows dimeric compounds to form via the benzyl ring and this limits the number of possible isomers (Supplementary Fig. S15). Product profiles for the hydroxycinnamates are much more complex compared to the profile for OH-dilignol (Fig. 3) and the complexity increase with the decrease in methoxy groups on the aromatic ring. The methoxy groups may cause sterical hindrance during radical driven propagation and therefore less products are formed after reaction on sinapic acid compared to *p*-coumaric acid.

## Discussion

OH-dilignol was chosen based on its similar structure to hydroxycinammates and higher degree of polymerization in order to have a substrate mimicking lignin. Despite the similar values of K_m_ (Table 1) the V_max_ was lower causing the apparent specific laccase activity for OH-dilignol (Table 2) to be lower than on the hydroxycinnamates. This difference could be related to resonance stabilization where the OH-dilignol might be capable of forming a more stable radical than the hydroxycinnamates^27^. Due to a lack in conjugated double bonds between the phenoxyl groups and the rest of the molecule the radical stays longer on the phenoxyl group before it reacts further. The analytical method does not allow the observation of radical formation and hence does not allow for interpretation of oxidation until the radical has reacted to deplete the substrate. On that notion it could be speculated that the laccases are equally efficient in oxidizing the OH-dilignol as they are in oxidizing the methoxylated hydroxycinnamates. The reaction of OH-dilignol with laccase showed only two isomers of the dimer (Fig. 3 panel g and h and Supplementary Figs S10 and S11) as a result of the lack of conjugation. The formation of the dimers is most likely linked via the 5−5′ position because there is a preference for formation linked in the *ortho* or *para* position which in this case only allows for 5−5′ linkage^26^ (possible structures are reported in Supplementary Fig. S15). These linkage preferences also explain why the complexity of the product profiles increased with the decrease in methoxylation on the aromatic ring. The higher the number of methoxylated groups, the higher the stereo-chemical hindrance and hence less products are formed.

Importantly, increasing number of methoxylations on the substrate molecule resulted in higher affinity by the laccases. For both Tv and Gl laccases the enzyme’s affinity follows sinapic acid ≥ ferulic acid > *p*-coumaric acid (Table 1), which is in accordance with hydroxycinnamates antioxidant power and other studies^19,28^. The preference for oxidizing the methoxylated substrates was not only seen with these simple monomeric molecules but was also found in pretreated lignocellulosic materials^29^.

The two different enzymes, Tv and Gl laccase, were applied at two different degrees of purity. The HPLC-MS showed that it was possible to monitor and quantify reaction parameters for both enzymes despite the differences in purity, concentration and potentially interfering compounds. In order to overcome the differences in purity and to allow direct comparison between the two enzymes the laccase activity was measured on syringaldazine and used to dose the two enzymes at the same level. The normalization against syringaldazine eliminated any differences in protein concentration and hence dosages could be based on addition of a fixed amount of activity rather than a fixed protein concentration. In this way a direct comparison between the two different enzymes was possible.

In order to obtain reliable quantification by LC-MS comparable conditions between calibration standards and unknown samples should be met. In this work, similar conditions were achieved by applying pure and simple reaction mixtures with little ionic strength thereby keeping the total number of ions low. In situation where differences between samples and calibration standards become too large the quantitative approach may not apply. Hence, an individual evaluation should be conducted in each situation before engaging in a new setup and there may be scenarios where the LC-MS methodology is not applicable.

Our results showed that the HPLC-MS can be used as a highly suitable assay for studying laccase activity and it has several advantages compared to more traditional methods based on change in UV absorbance^12,19,30^. The HPLC-MS allows fast (few minutes retention time), online analysis in real time, meaning that the reaction is monitored while it proceeds with minimal disturbance. A negligible amount of sample volume (2 *μ*L) is drawn from the reaction at any given time point and the enzyme reaction is immediately quenched when the sample is injected into the flow path (mobile phase, pH 2). This type of analysis is also highly descriptive of the reaction both in terms of product profiling with mass detection and also in relation to maintaining the integrity of the sample when no artifacts are introduced by extensive sample handling such as heat inactivation. HPLC-MS also offers high sensitivity with enzyme concentrations in *μ*U scale and substrate concentration in sub-pmol scale compared to UV signals reported in the nmol scale^12^. Comparison between MS (in Multiple Reaction Monitoring-mode) and UV signals directly measured in this work showed that the lower limit for substrate detection is approximately 0.04 pmol on the MS versus approximately 2 pmol in UV signal. One of the explanations for this high sensitivity is low levels of noise on the MS in Multiple Reaction Monitoring-mode. With respect to assay specificity the method allows to distinguish laccase from peroxidase activity because the latter requires an additional co-substrate in the form of H_2_O_2_. Polyphenol oxidase may also respond in this type of assay and in case of doubt substrates more specific for polyphenol oxidase might need to be included.

The original hypothesis that laccases have different reaction kinetics on the three lignin units has been investigated and the work shows that laccases indeed have different preferences for oxidizing structures that resemble lignin - the low solubility of lignin and the apparent dependency of laccase kinetics of the availability of reactive phenol-hydroxyls in larger lignin structure is a separate issue^18^. This work also shows that hydroxycinnamates are reliable models for lignin-derived phenols and smaller lignin polymeric moieties and that the hydroxycinnamates are excellent candidates as model substrates for laccases.

## Methods

### Materials

Sinapic acid, ferulic acid, *p*-coumaric acid and all the chemicals used in the work were purchased from Sigma-Aldrich (Steinheim, Germany). OH-dilignol was a kind gift from Prof. Dr. Carsten Bolm, Institute of Organic Chemistry in RWTH Aachen University. Laccase from *Trametes versicolor* (Tv) was purchased from Sigma-Aldrich (Steinheim, Germany) and the *Ganoderma lucidum* (Gl) laccase was produced in house using *Pichia pastoris* as heterogeneous expression system. The construct containing the gene encoding Gl (pML *α*-LacGL1) was produced similar to previous work^17^, however in this work an enzyme without His-tag was used. A 5 L scale production of recombinant laccase in *P. pastoris* was performed according to Silva *et al*.^31^. In order to improve enzyme’s stability the methanol Fed-Batch phase was carried out at 20 °C. The total time for the fermentation process was 112 h. Laccase enriched fermentation broth was recovered by centrifugation at 5300 × g 5 °C for 1 h and the supernatant was subjected to sterile filtration and concentrated by ultrafiltration, using a cross-flow bioreactor system with a 10 kDa cutoff membrane (Millipore, Sartorius, Denmark), as described by Silva *et al*.^31^. The enzyme aliquots were stored at −80 °C with the addition of 20% (w/v) glycerol. In the present work Gl laccase was used directly after concentration of the fermentation supernatant. No further purification steps were performed.

### Laccase activity assay

Activity of laccase was assessed by monitoring the oxidation of syringaldazine (SGA) at 530 nm (*ε* = 6.5 × 10^4^ M^−1^ cm^−1^). The assay reaction mixture contained 25 *μ*M syringaldazine, 10% ethanol, 25 mM sodium acetate pH 5.0 and a proper amount of enzyme. Syringaldazine oxidation was monitored at 25 °C for 20 minutes. Enzyme activity was expressed in units: one International Unit (U) was defined as the amount of enzyme required to catalyse the conversion of 1 *μ*mol of substrate (syringaldazine) per minute under the assay reaction conditions. SGA activity was used in this work as measure of enzyme dose to overcome the differences in enzyme purity. Therefore Tv and Gl laccases were dosed on the same value of SGA activity in the HPLC-MS reactions diluting them in 12.5 mM sodium acetate buffer pH 5.

### HPLC-MS screening method

Laccase oxidation of sinapic acid, ferulic acid, *p*-coumaric acid and OH-dilignol and formation of products were assessed using liquid chromatography and mass spectroscopy (HPLC-MS). The reaction mixture (reaction volume of 500 *μ*L) contained 50 *μ*M of substrate in 12.5 mM sodium acetate buffer pH 5 and an appropriate dose of enzyme depending on the substrate analysed, i.e. 2.5 *μ*U for sinapic acid and ferulic acid, 220 *μ*U for *p*-coumaric acid and 5 *μ*U for OH-dilignol (where needed the enzymes were diluted in 12.5 mM sodium acetate buffer pH 5). The samples were incubated in the HPLC autosampler at 30 °C. Two *μ*L of reaction mixture were injected onto a Hypersil Gold Phenyl column (150 mm × 2.1 mm; 3 *μ*m, Thermo Fisher Scientific, Waltham, MA, USA). The chromatography was performed on a Dionex UltiMate 3000 UPLC (Thermo Fischer Scientific, Sunnyvale, CA, USA) at 0.4 mL min^−1^ at 40 °C with a three-eluent system with eluent A 0.1% formic acid in water, eluent B acetonitrile and eluent C water. The elution was performed as follow (time indicated in min): 0, 10% A 0% B 90% C; 0–15, linear gradient to 10% A 90% B 0% C; 15–20, isocratic 10% A 90% B 0% C; 20–25, isocratic 10% A 0% B 90% C. The HPLC was connected to an ESI-iontrap (model Amazon SL from Bruker Daltonics, Bremen, Germany) and the electrospray was operated in negative ultra scan mode using a target mass of 300 *m/z* for the three acid and a target mass of 400 *m/z* for OH-dilignol. A scan range from 50 to 2200 *m/z* was selected and capillary voltage at 4.5 kV, end plate offset 0.5 kV, nebulizer pressure at 3.0 bar, dry gas flow at 12.0 L min^−1^, and dry gas temperature at 280 °C were used.

### HPLC-MS kinetic method

Laccase kinetic on sinapic acid, ferulic acid, *p*-coumaric acid and OH-dilignol was assayed with liquid chromatography and mass spectroscopy (HPLC-MS) with a set-up similar to the one described above, however with minor adjustments to accommodate short time intervals between sampling. The reaction (reaction volume of 500 *μ*L) was performed using different concentrations of the substrate ranging from 2 *μ*M to 50 *μ*M, 12.5 mM sodium acetate buffer pH 5 and three different dosage of enzyme: 0.625, 1.25 and 2.5 *μ*U for sinapic acid and ferulic acid; 55, 110 and 220 *μ*U for *p*-coumaric acid; and 1.25, 2.5 and 5 *μ*U for OH-dilignol (where needed the enzymes were diluted in 12.5 mM sodium acetate buffer pH 5). Two *μ*L of the reaction mixture were injected onto a Hypersil Gold Phenyl column. For reaction performed at 2 and 4 *μ*M substrate concentration, 5 *μ*L sample were injected to have sufficient signal intensity. This was taken into account during quantification. The chromatography was performed at 0.4 mL min^−1^ at 40 °C on a three-eluent system with eluent A 0.1% formic acid in water, eluent B acetonitrile and eluent C water. The elution profile was performed as follow (time indicated in min): 0–1.5, isocratic 10% A, 30% B 60% C; 1.5–4, step gradient to 10% A 90% B 0%; 4–6, isocratic 10% A, 30% B 60% C. The HPLC was connected to an ESI-iontrap and the electrospray was operated in negative ultra scan with Multiple Reaction Monitoring (MRM) mode using a target mass of 200 *m/z* for the acids and of 300 *m/z* for OH-dilignol. MRM mode was chosen to selectively follow only substrate depletion. MRM ion precursor were chosen according to the [M − H]^−^ value for the different substrates assayed, i.e. *m/z* 222.83, *m/z* 192.74, *m/z* 162.79 and *m/z* 319.10, for sinapic acid, ferulic acid, *p*-coumaric acid and OH-dilignol respectively. 100% amplitude for reaction was selected in order to have fragmentation of the ion precursor examined. Capillary voltage at 4.5 kV, end plate offset 0.5 kV, nebulizer pressure at 3.0 bar, dry gas flow at 12.0 L min^−1^, and dry gas temperature at 280 °C were used. Standards of each substrate at different concentration, ranging from 1 to 100 *μ*M were also analysed as external standards with the same method for calibration.

### Quantification

Quantification of the precursor ion was performed using Bruker Compass QuantAnalysis software (Bruker Daltonik GmbH), defining an individual method for each substrate. All ions were observed as [M − H]^−^. Sinapic acid quantification was performed by defining an Extracted Ion Chromatogram (EIC) on MSn of *m/z* 222.8, masses *m/z* 222.75, *m/z* 207.72, *m/z* 178.75 and *m/z* 163.78 with a width of ± 0.5, retention time 1.4 min with a window of 0.2 min. Ferulic acid quantification was performed by defining an EIC on MSn of *m/z* 192.7, masses *m/z* 192.72, *m/z* 177.71, *m/z* 148.80 and *m/z* 133.85 with a width of ± 0.5, retention time 1.4 min with a window of 0.2 min. *p*-coumaric acid quantification was performed by defining an EIC on MSn of *m/z* 162.8, masses *m/z* 162.79 and *m/z* 119.00 with a width of ± 0.5, retention time 1.4 min with a window of 0.2 min. OH-dilignol quantification was performed by defining an EIC on MSn of *m/z* 319.1, masses *m/z* 319.10, *m/z* 270.75 and *m/z* 194.77 with a width of ± 0.5, retention time 1.7 min with a window of 0.2 min. In all the cases peak detection was done using algorithm version 2.1, S/N threshold 1, area threshold 0.1, intensity threshold 0.1, skim ratio 0.1 and smoothing width 1. Calibration curve was performed using 9 levels of concentrations and fitting the data with a quadratic curve (see Supplementary Figs S16 and S17 to see how the different masses were chosen and how the standard curves looked).

### Kinetic parameters determination

Kinetic parameters V_max_ and K_m_ were obtained using Hane linearization of the Michaelis-Menten curve for all the different enzyme dosages assessed. Apparent specific activity was determined by normalizing V_max_ over the highest value of enzyme dosage used. The normalization was performed using 2.5 *μ*U for sinapic acid and ferulic acid, 220 *μ*U for *p*-coumaric acid and 5 *μ*U for OH-dilignol. Apparent catalytic activity was determined by dividing the apparent specific activity by K_m_.

### Statistical analysis

One-way ANOVA for determination of statistical significance was made in Minitab 18 (Minitab Inc., State College, PA, USA) using Tukey’s test with a pooled standard deviation. Statistical significance was established at p ≤ 0.05.

## Electronic supplementary material

Supplementary information
