# Supplementary material for: Multiple Reaction Monitoring for quantitative laccase kinetics by LC-MS
Source: Sci Rep. 2018 May 25;8:8114. doi: 10.1038/s41598-018-26523-0 (PMC5970232; doi:10.1038/s41598-018-26523-0)
Supplement: Supplementary file 1 — Supplementary information [file 41598_2018_26523_MOESM1_ESM.pdf]

Multiple Reaction Monitoring for quantitative laccase kinetics by LC-MS / Supplementary information

Valentina Perna[1], Jane W. Agger[1,*], Jesper Holck[1], and Anne S. Meyer[1]

[1]Center for BioProcess Engineering, Department of Chemical and Biochemical Engineering, Technical University of Denmark, Kgs. Lyngby, 2800, Denmark
[*]jaag@kt.dtu.dk

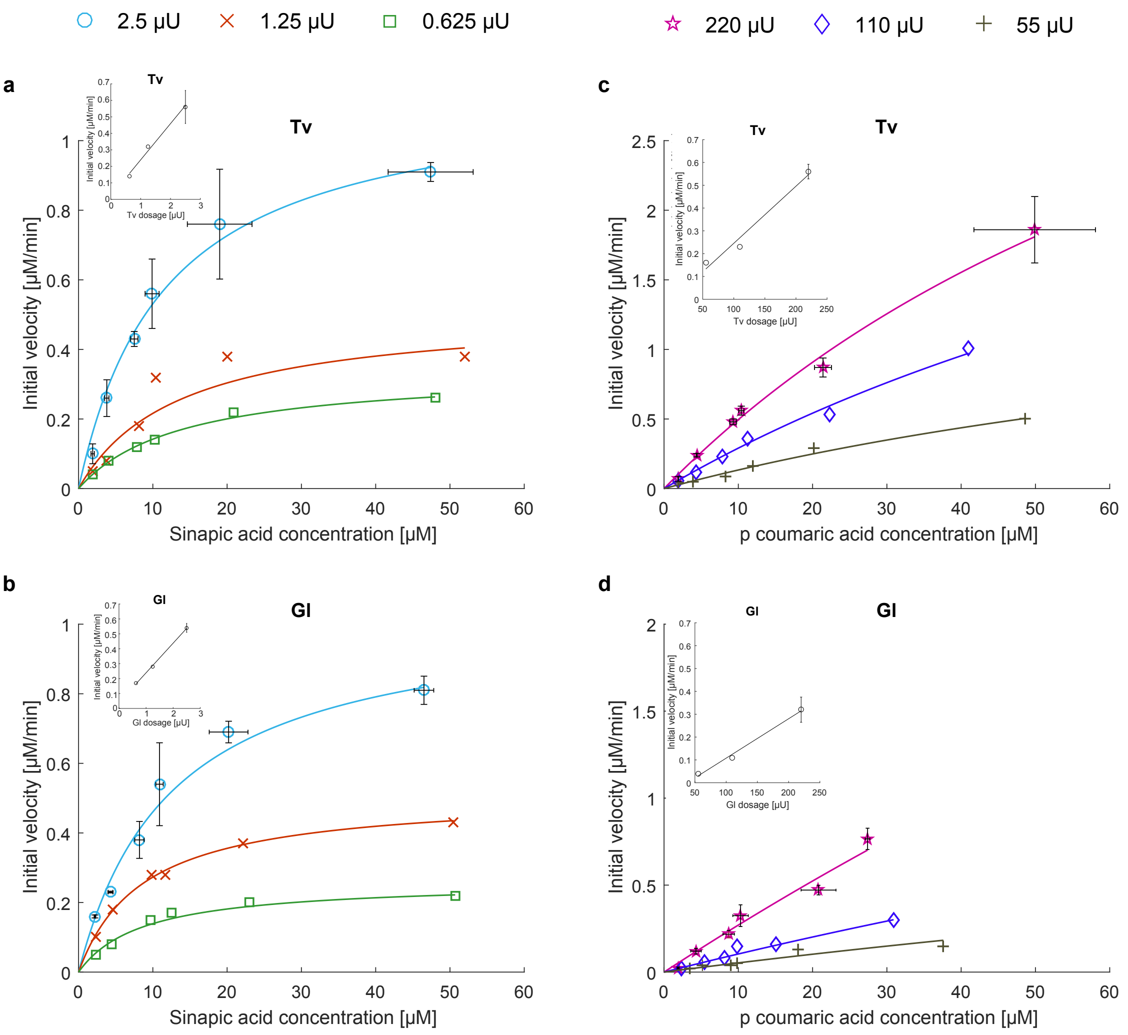

Figure S1. Michaelis-Menten curves for *Trametes versicolor* and *Ganoderma lucidum* laccase on sinapic acid (a, b) and *p*-coumaric acid (c, d). Three different enzyme dosages are shown: for sinapic acid (a, b): 0.625 μU (green open square), 1.25 μU (red cross) and 2.5 μU (blue open circle). For *p*-coumaric acid (c, d): 55 μU (grey cross), 110 μU (dark blue open diamond) and 220 μU (purple open star). Dose response at 10 μM substrate concentration is shown in the inset. For the highest enzyme dose standard deviation are shown. Y-axis is optimized in order to have a optimal view of the curves.

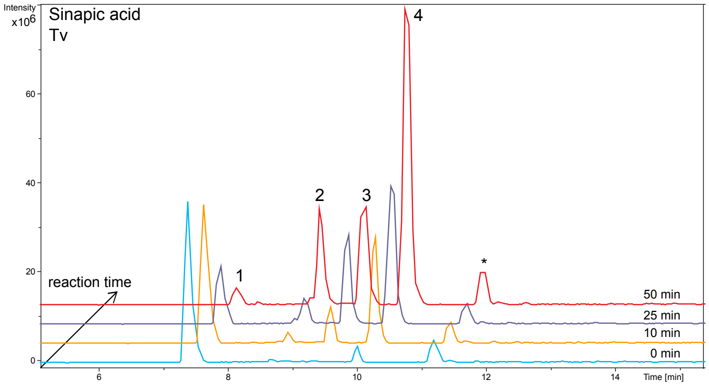
Intensity
x10^6
Sinapic acid
Tv
reaction time
1
2
3
4
*
50 min
25 min
10 min
0 min
Time [min]

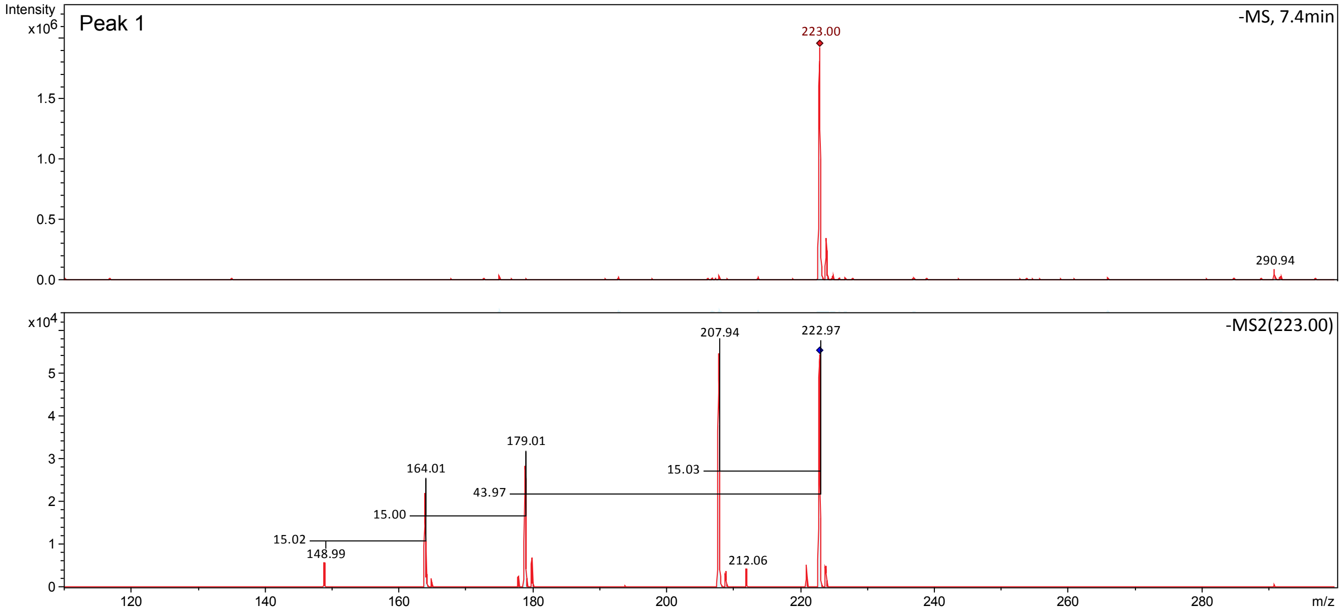
Intensity
x10^6
Peak 1
-MS, 7.4min
223.00
290.94
x10^4
-MS2(223.00)
207.94
222.97
179.01
164.01
15.03
43.97
15.00
15.02
148.99
212.06
m/z

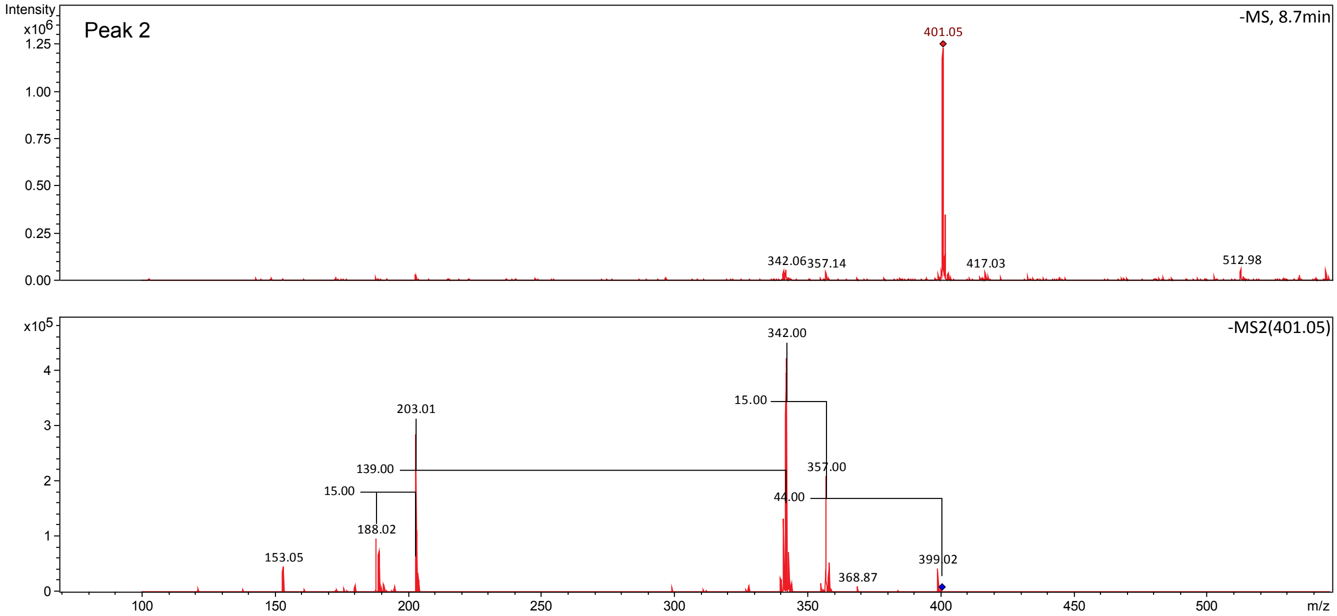
Intensity
x10^6
Peak 2
-MS, 8.7min
401.05
342.06
357.14
417.03
512.98
x10^5
-MS2(401.05)
342.00
203.01
15.00
139.00
357.00
44.00
15.00
188.02
153.05
399.02
368.87
m/z

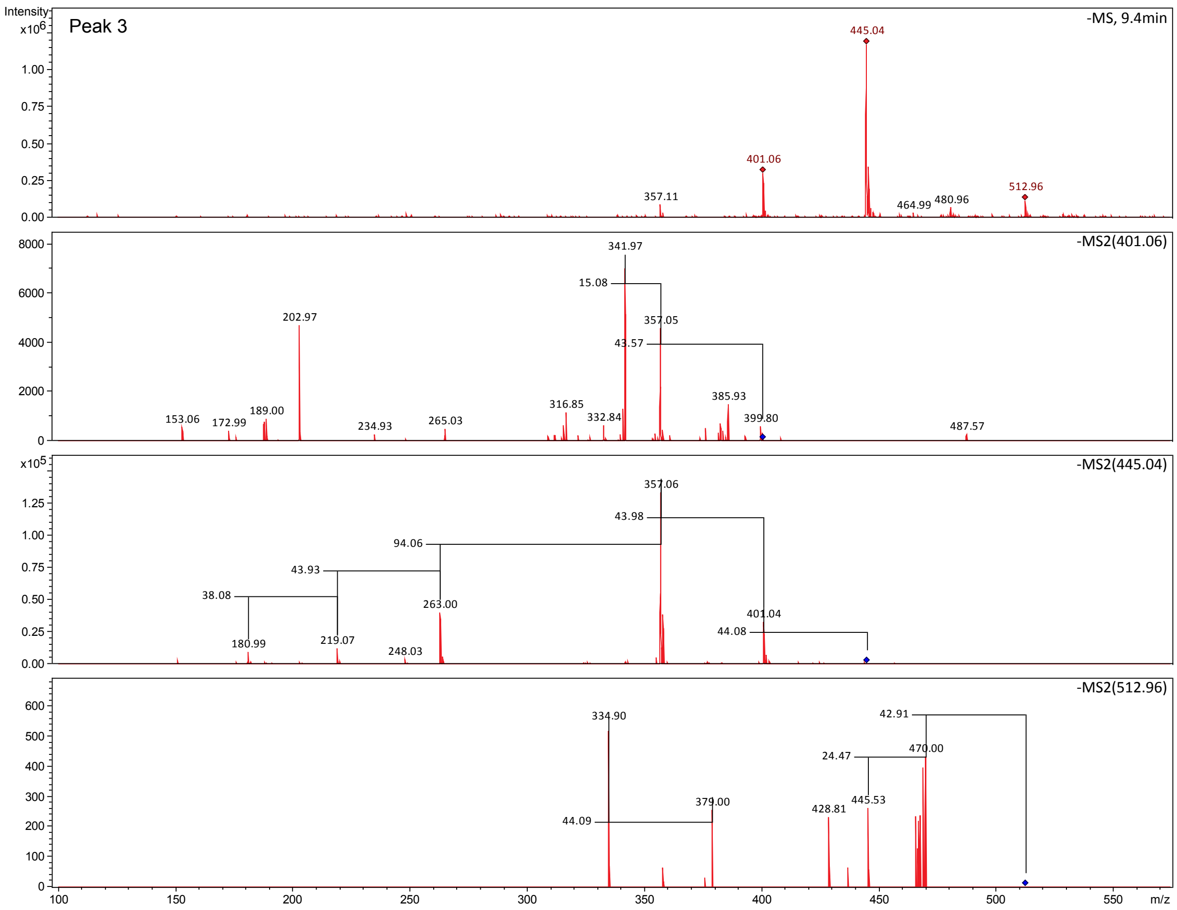
Intensity
x10^6
Peak 3
-MS, 9.4min
445.04
401.06
512.96
357.11
464.99
480.96
-MS2(401.06)
341.97
15.08
357.05
202.97
43.57
385.93
316.85
189.00
332.84
399.80
153.06
172.99
234.93
265.03
487.57
x10^5
-MS2(445.04)
357.06
43.98
94.06
43.93
38.08
263.00
401.04
44.08
180.99
219.07
248.03
-MS2(512.96)
334.90
42.91
470.00
24.47
379.00
445.53
428.81
44.09
m/z

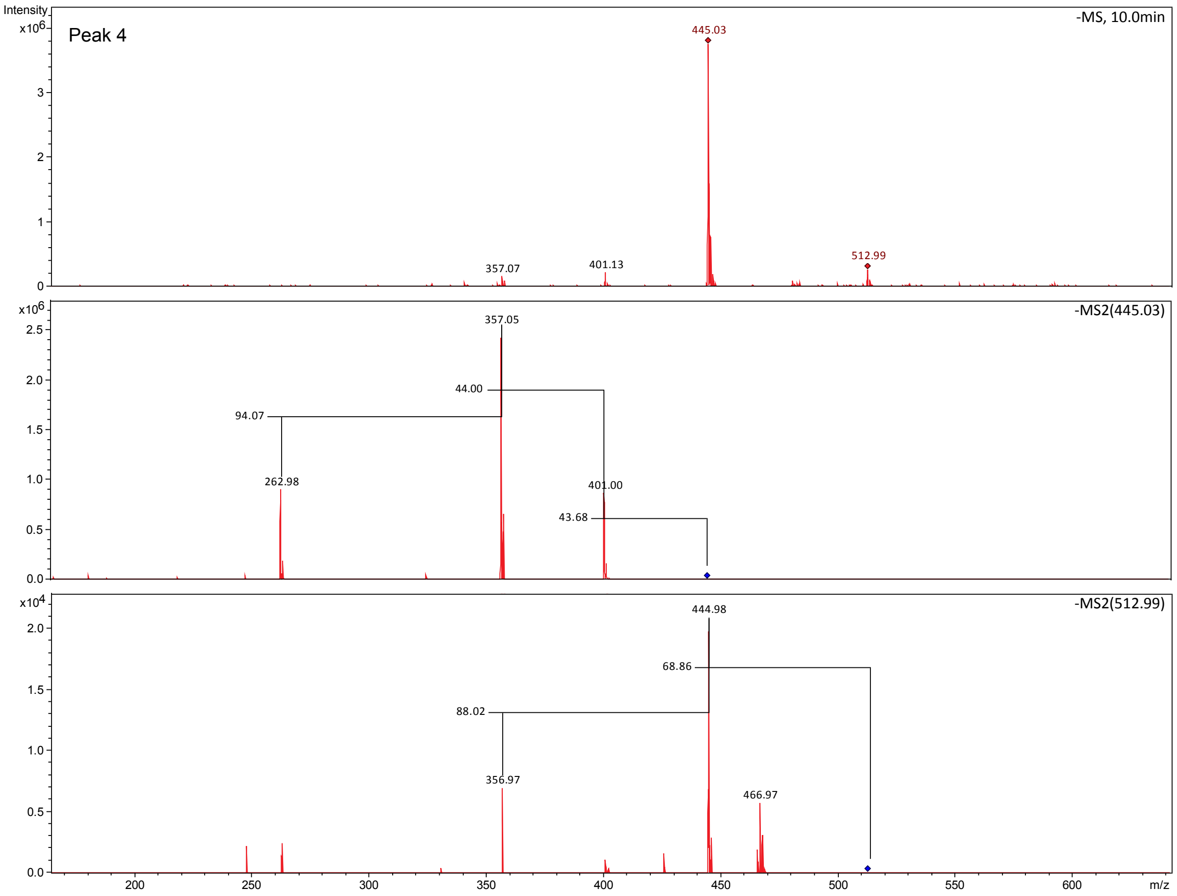
Intensity
x10^6
Peak 4
-MS, 10.0min
445.03
512.99
357.07
401.13
x10^6
-MS2(445.03)
357.05
44.00
94.07
262.98
401.00
43.68
x10^4
-MS2(512.99)
444.98
68.86
88.02
356.97
466.97
m/z

Figure S2. Evolution profile of sinapic acid oxidation by *Trametes versicolor* laccase. LC chromatograms at different reaction times are shown: 0 minutes (light blue), 10 minutes (yellow), 25 minutes (violet) and 50 minutes (red). MS and MS/MS spectra for each peak are also shown. All ions are observed as $[M\text{-}H]^-$. As reaction time is increasing substrate peak (peak 1) is decreasing in intensity, while product peaks (peak 2, 3 and 4) are increasing in intensity, meaning that the substrate is being consumed and the products formed. Major losses during fragmentation are indicated on the MS/MS spectra when relevant. Product peaks are not always characterized by one single mass, but two or more different compounds can be found in each peak. Whether it is a result of co-elution of two or more different products or chemical degradation has not be investigated. Sinapic acid *m/z* 223 elutes at a retention time (RT) of 7.4 min (peak 1). Three products can be distinguished already after 10 minutes of reaction. Two isomers of the dimer *m/z* 445.05 are eluting at RT 9.4 and 10 min (peak 3 and 4). A sodium formate adduct (+ 68) of the dimer can be also found in peak 3 and 4 characterized by *m/z* 513. Peak 2 RT 8.7 min is characterized by *m/z* 401.05 which correspond to a dimer with the loss of 44, most likely the loss of a carboxilic group. Peak * RT 11.3 min, containing *m/z* 293.13 and *m/z* 361.13, where the latter is a sodium formate adduct of *m/z* 293.13, was also found by Petrucci *et al.*[1] (corresponding MS spectra is shown in Supplementary Figure S10). It is, most likely, an impurity of LC-MS eluent system since it is present also in the blank run. Suggested chemical structure for the major products are shown in Supplementary Figure S12.

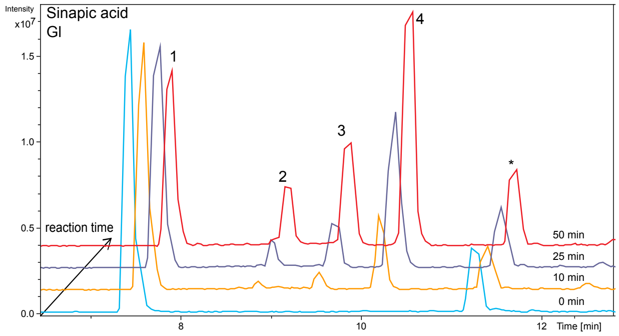
Intensity x10^7
Sinapic acid
GI
1
2
3
4
*
reaction time
50 min
25 min
10 min
0 min
Time [min]

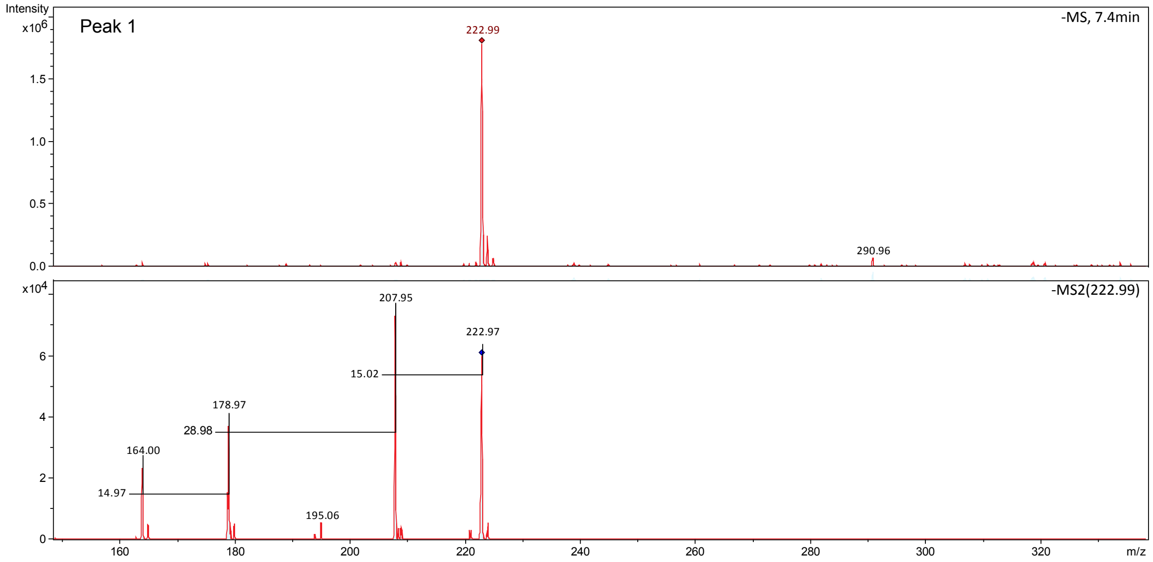
Intensity x10^6
Peak 1
-MS, 7.4min
222.99
290.96
-MS2(222.99)
207.95
222.97
15.02
178.97
28.98
164.00
14.97
195.06
m/z

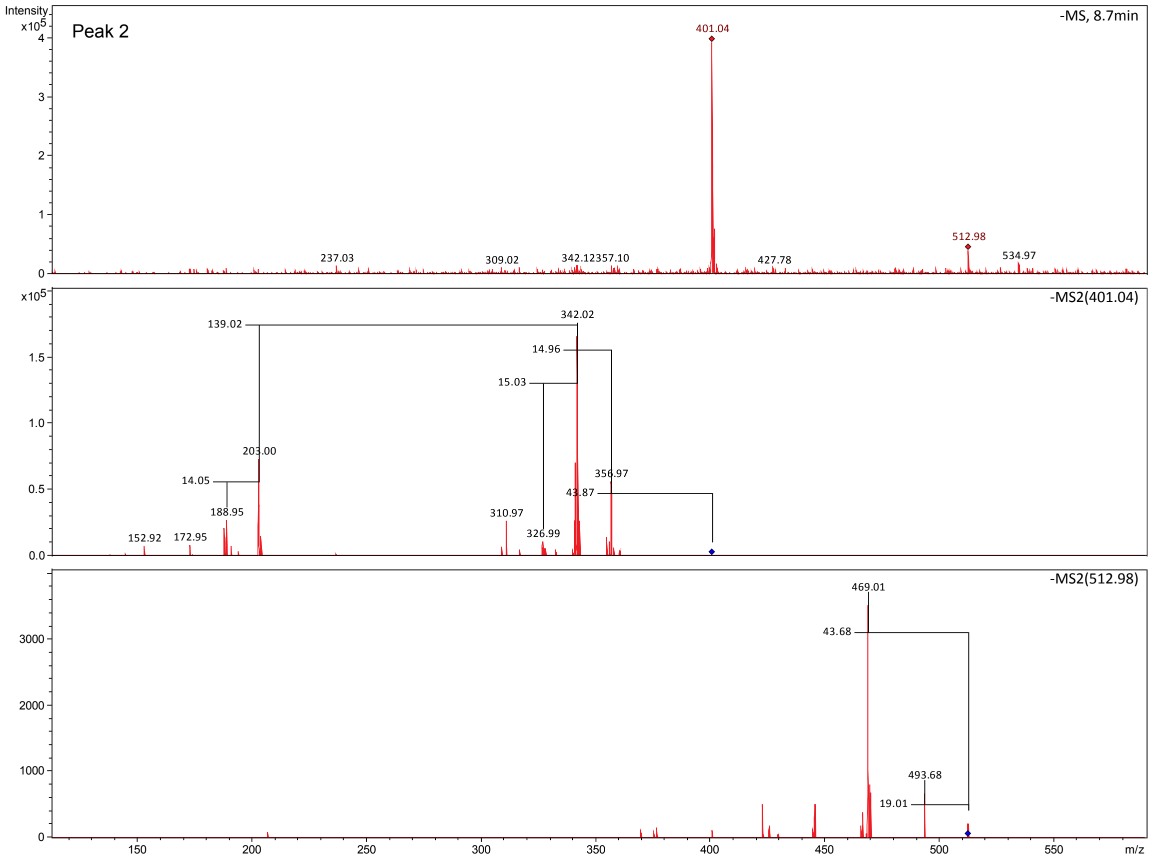
Intensity x10^5
Peak 2
-MS, 8.7min
401.04
512.98
534.97
237.03
309.02
342.12357.10
427.78
-MS2(401.04)
342.02
139.02
14.96
15.03
203.00
14.05
188.95
356.97
43.87
310.97
326.99
152.92
172.95
-MS2(512.98)
469.01
43.68
493.68
19.01
m/z

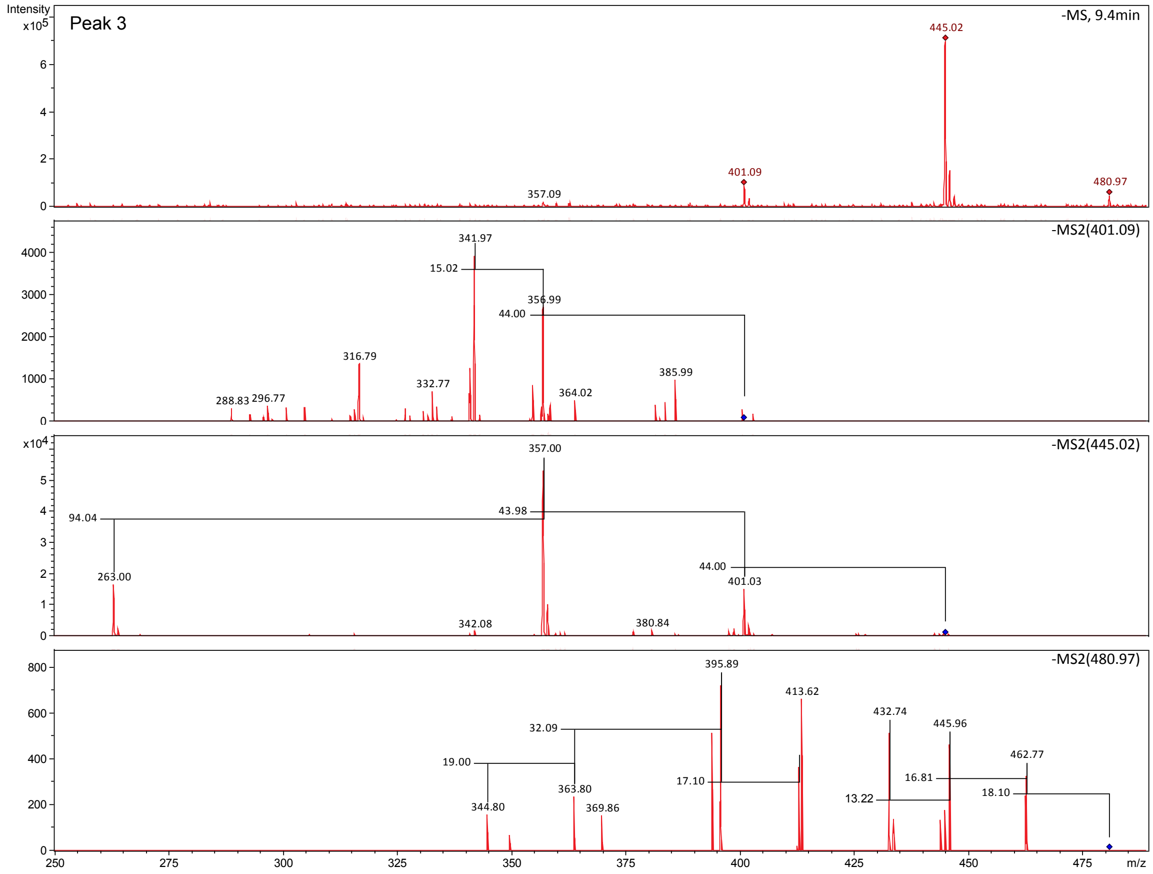
Intensity x10^5
Peak 3
-MS, 9.4min
445.02
401.09
357.09
480.97
-MS2(401.09)
341.97
15.02
356.99
44.00
316.79
332.77
385.99
364.02
288.83
296.77
x10^4
-MS2(445.02)
357.00
94.04
43.98
44.00
401.03
263.00
342.08
380.84
-MS2(480.97)
395.89
413.62
432.74
445.96
462.77
32.09
19.00
363.80
369.86
344.80
17.10
16.81
18.10
13.22
m/z

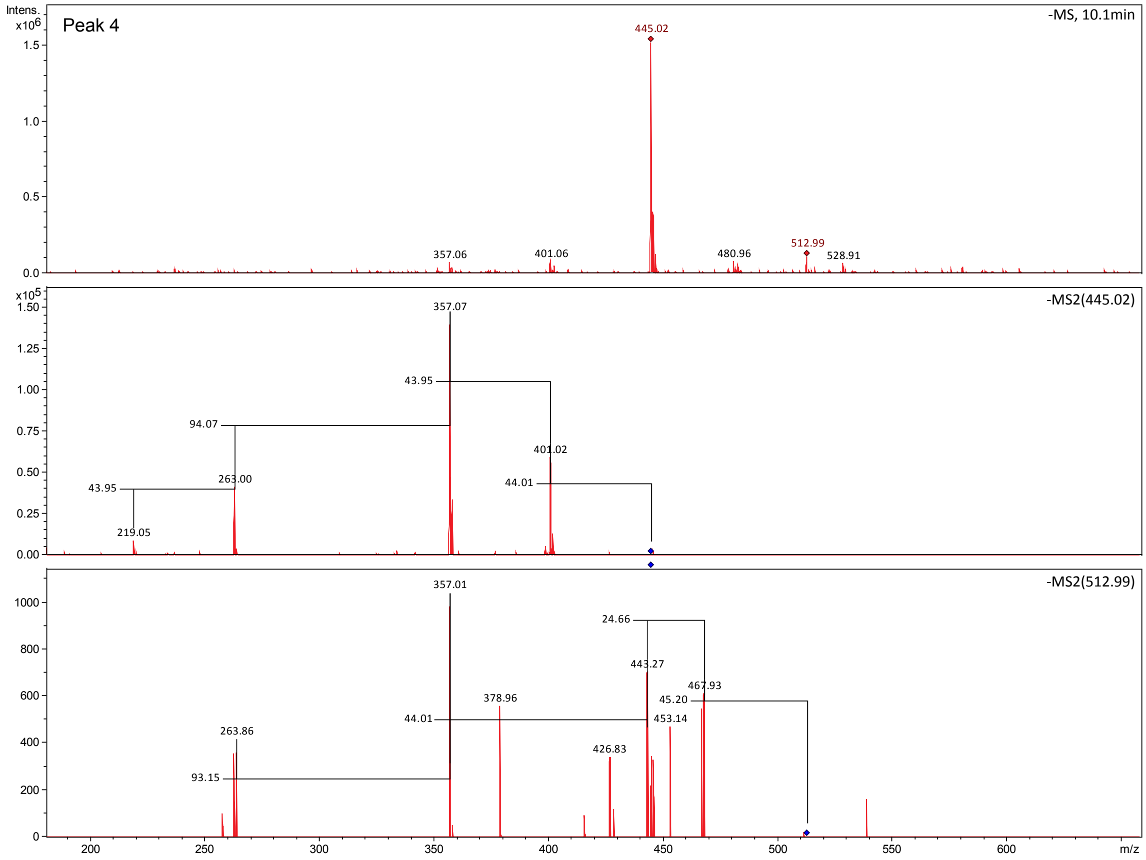
Intens. x10^6
Peak 4
-MS, 10.1min
445.02
357.06
401.06
480.96
512.99
528.91
x10^5
-MS2(445.02)
357.07
43.95
94.07
401.02
44.01
263.00
43.95
219.05
-MS2(512.99)
357.01
24.66
443.27
467.93
45.20
378.96
453.14
44.01
426.83
263.86
93.15
m/z

Figure S4. Evolution profile of sinapic acid oxidation by *Ganoderma lucidum* laccase. LC chromatograms at different reaction times are shown: 0 minutes (light blue), 10 minutes (yellow), 25 minutes (violet) and 50 minutes (red). MS and MS/MS spectra for each peak are also shown. All ions are observed as $[M\text{-}H]^-$. As reaction time is increasing substrate peak (peak 1) is decreasing in intensity, while product peaks (peak 2, 3 and 4) are increasing in intensity, meaning that the substrate is being consumed and the products formed. Major losses during fragmentation are indicated on the MS/MS spectra when relevant. Product peaks are not always characterized by one single mass, but two or more different compound can be found in each peak. Whether it is a result of co-elution of two or more different product or chemical degradation has not be investigated. Sinapic acid *m/z* 223 elutes from the chromatographic column at a retention time (RT) 7.4 min (peak 1). Three product can be distinguished already after 10 minutes of reaction. Two isomers of the dimer *m/z* 445.05 are eluted at RT 9.4 and 10 min (peak 3 and 4). Three isomers of the decarboxylated dimer (loss of 44) characterized by *m/z* 401.06 are found in peak 2, 3 and 4. A sodium formate adduct (+ 68) of the dimer can be also found in peak 2 and 4 characterized by *m/z* 513. Peak 3 contains masses that could not be immediately resolved to a proposed structure. Peak * RT 11.3 min, containing *m/z* 293.13 and *m/z* 361.13, where the latter is a sodium formate adduct of *m/z* 293.13, was also found by Petrucci *et al.*[1] (corresponding MS spectra is shown in Supplementary Figure S11). It is, most likely, an impurity of LC-MS eluent system since it is present also in the blank run. Suggested chemical structure for the major products are shown in Supplementary Figure S12.

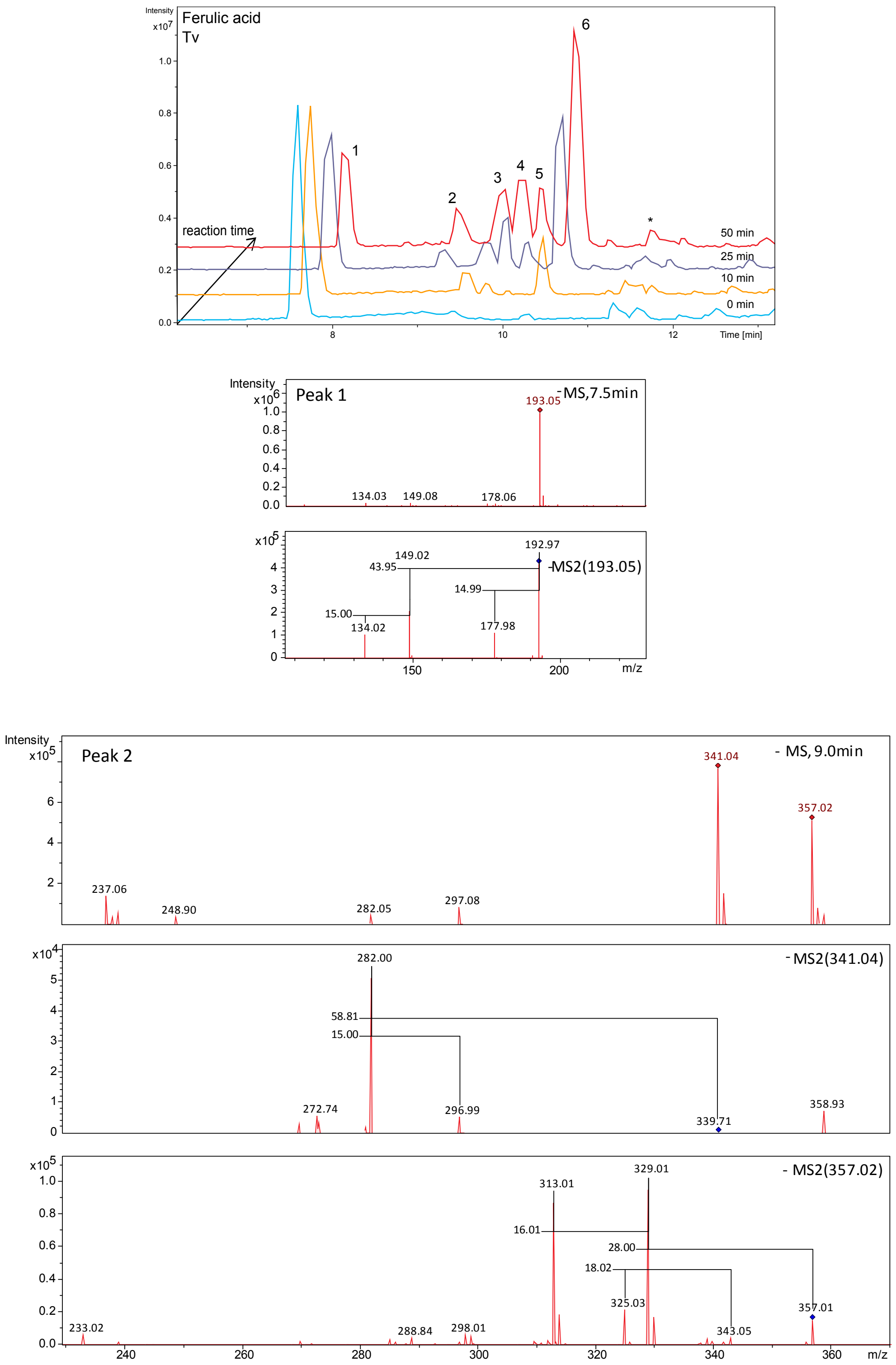
Ferulic acid
Tv
reaction time
50 min
25 min
10 min
0 min
Time [min]
Peak 1
-MS,7.5min
-MS2(193.05)
Peak 2
- MS, 9.0min
- MS2(341.04)
- MS2(357.02)
m/z

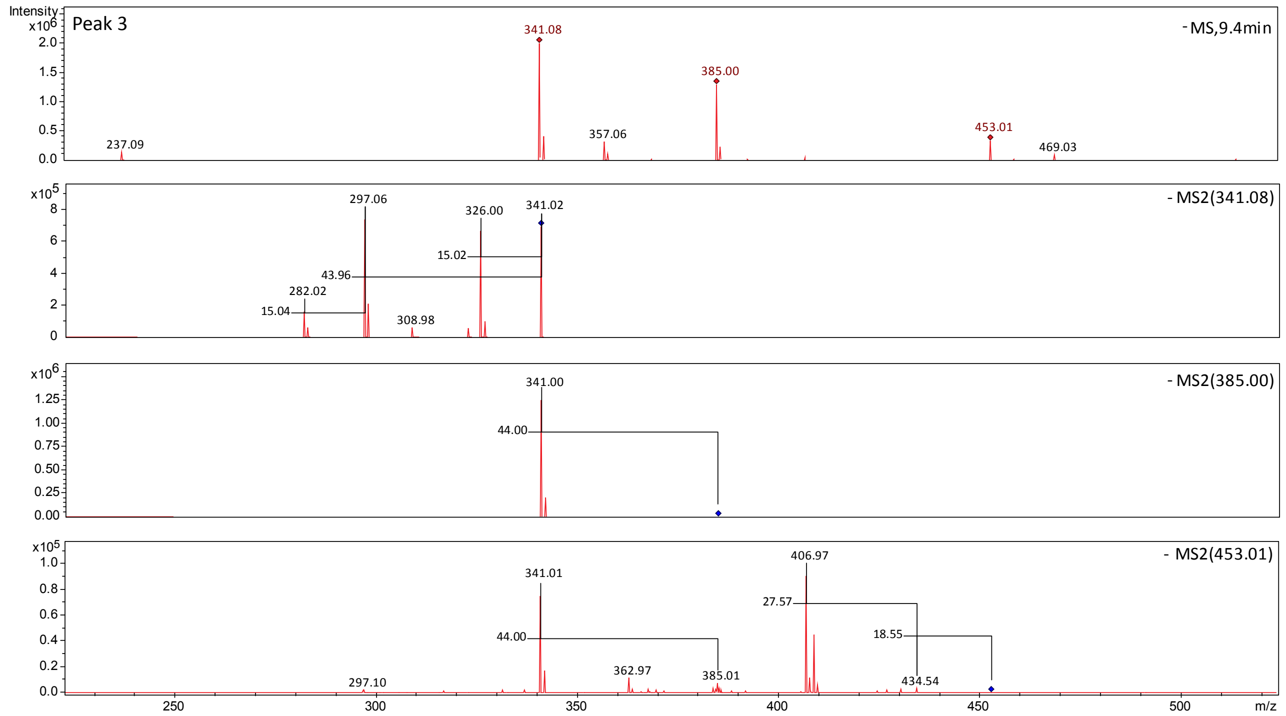
Peak 3
- MS,9.4min
- MS2(341.08)
- MS2(385.00)
- MS2(453.01)
Intensity
m/z

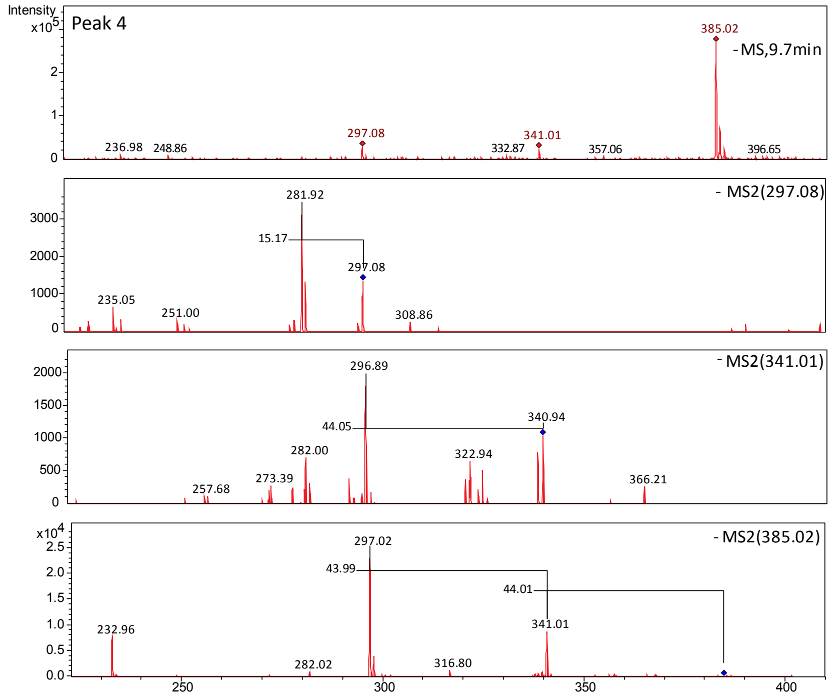
Peak 4
- MS,9.7min
- MS2(297.08)
- MS2(341.01)
- MS2(385.02)
Intensity
m/z

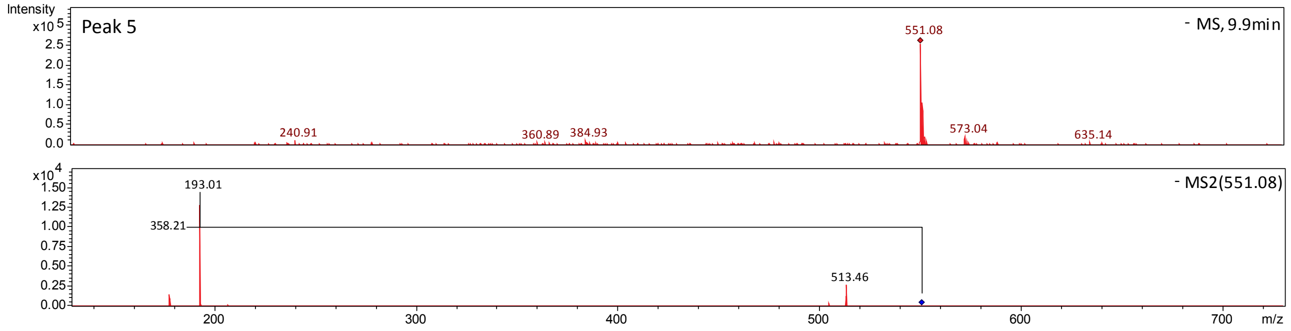
Peak 5
- MS, 9.9min
- MS2(551.08)
Intensity
m/z

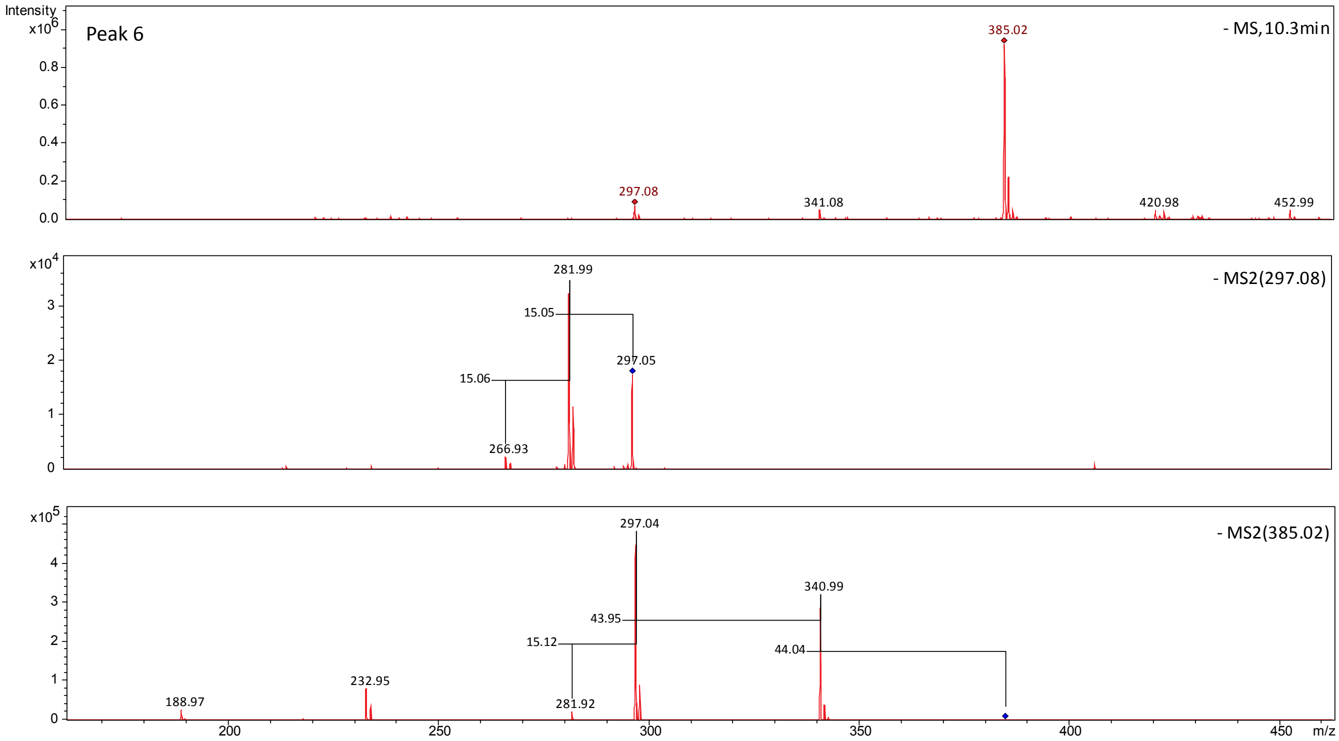

Figure S6. Evolution profile of ferulic acid oxidation by *Trametes versicolor* laccase. LC chromatograms at different reaction times are shown: 0 minutes (light blue), 10 minutes (yellow), 25 minutes (violet) and 50 minutes (red). MS and MS/MS spectra for each peak are also shown. All ions are observed as $[M-H]^-$. As reaction time is increasing substrate peak (peak 1) is decreasing in intensity, while product peaks (peaks 2-6) are increasing in intensity, meaning that the substrate is being consumed and the products formed. Major losses during fragmentation are indicated on the MS/MS spectra when relevant. Product peaks are not always characterized by one single mass, but two or more different ones elute at the same time. Whether it is a result of co-elution of two or more different product or chemical degradation has not be investigated. Ferulic acid is identified with the *m/z* 192.99 and a retention time (RT) of 7.6 min. After 10 minutes reaction time four different product peaks are present with RT 9.4, 9.7, 9.9 and 10.3 (peak 3, 4, 5 and 6), while a fifth product peak is appearing after 25 minutes at RT 8.9 min (peak 2). Three different isomers of ferulic acid dimer are present in peak 3, 4 and 6 characterized by *m/z* 385.02. The decarboxylated dimer *m/z* 341.06 (characterized by the loss of 44) is also present in three different isomeric forms (peak 2, 3 and 4). Peak 2 and 6 also contain, a compound with *m/z* 297.08 corresponding to a double decarboxylated dimer. A sodium formate adduct of the dimer can be found in peak 3 characterized by *m/z* 453. Peak 2 and 5 contain masses that could not be immediately resolved to a proposed structure. Peak * RT 11.3 min, containing *m/z* 293.13 and *m/z* 361.13, where the latter is a sodium formate adduct of *m/z* 293.13, was also found by Petrucci *et al.*[1] (corresponding MS spectra is shown in Supplementary Figure S10). It is, most likely, an impurity of LC-MS eluent system since it is present also in the blank run. Suggested chemical structure for the major products are shown in Supplementary Figure S13.

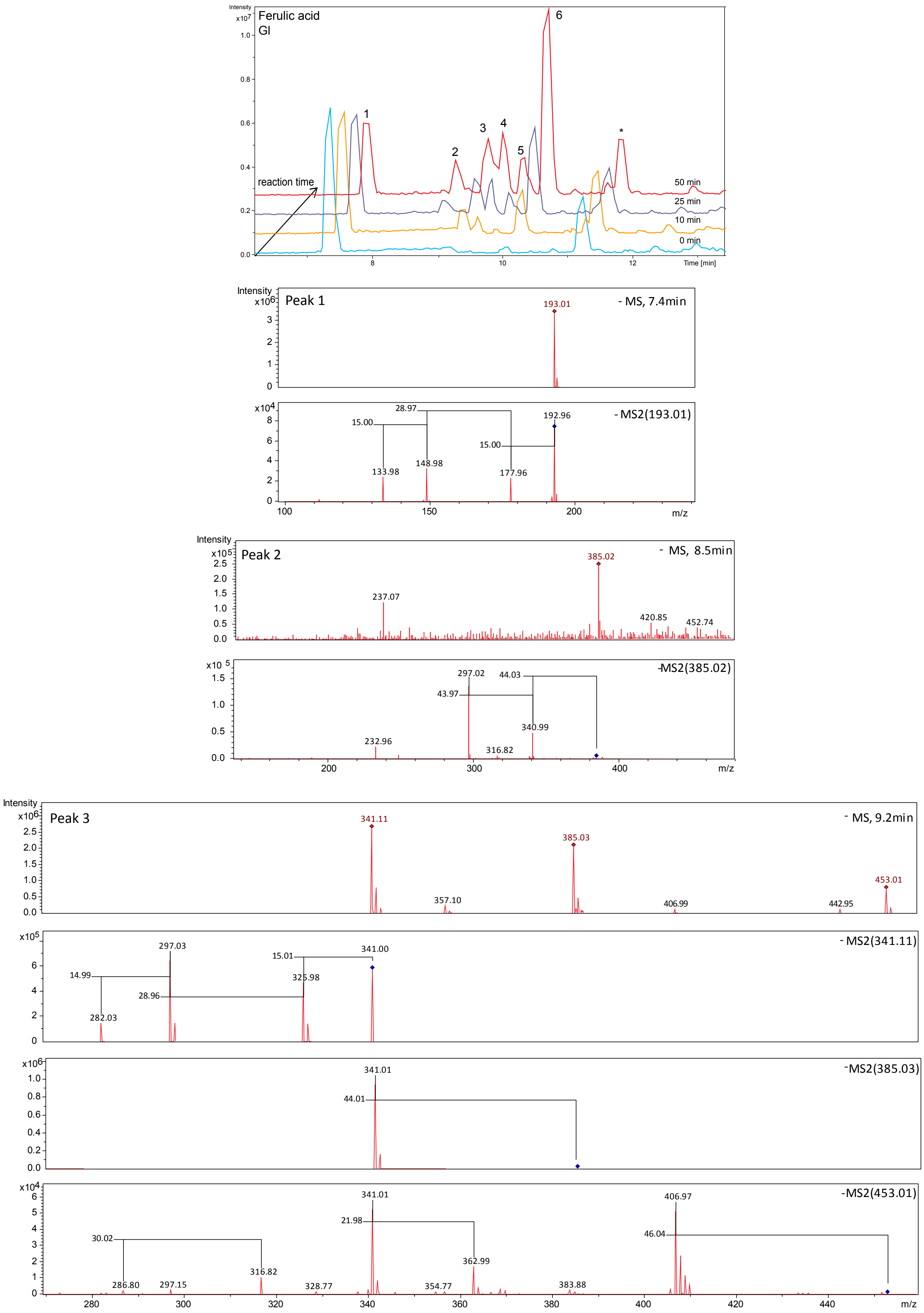

Intensity x10^7
Ferulic acid
GI
reaction time
1
2
3
4
5
6
*
50 min
25 min
10 min
0 min
Time [min]
Peak 1
- MS, 7.4min
193.01
- MS2(193.01)
28.97
15.00
192.96
15.00
148.98
133.98
177.96
m/z
Peak 2
- MS, 8.5min
385.02
237.07
420.85
452.74
-MS2(385.02)
297.02
44.03
43.97
340.99
232.96
316.82
Peak 3
- MS, 9.2min
341.11
385.03
453.01
357.10
406.99
442.95
- MS2(341.11)
297.03
15.01
341.00
14.99
325.98
28.96
282.03
-MS2(385.03)
341.01
44.01
-MS2(453.01)
341.01
406.97
21.98
46.04
30.02
362.99
316.82
286.80
297.15
328.77
354.77
383.88

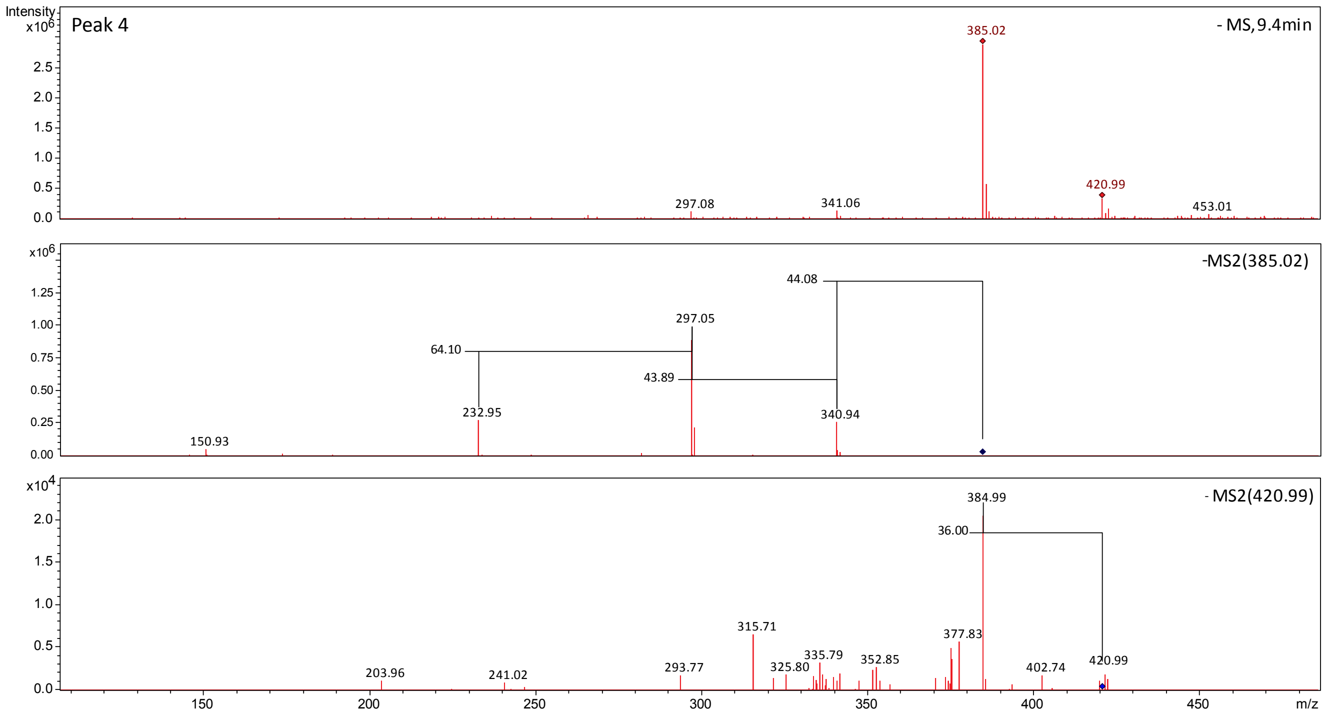
Intensity x10^6
Peak 4
- MS, 9.4min
385.02
420.99
297.08
341.06
453.01
-MS2(385.02)
44.08
297.05
64.10
43.89
232.95
340.94
150.93
- MS2(420.99)
384.99
36.00
315.71
377.83
335.79
325.80
352.85
402.74
420.99
293.77
203.96
241.02
m/z

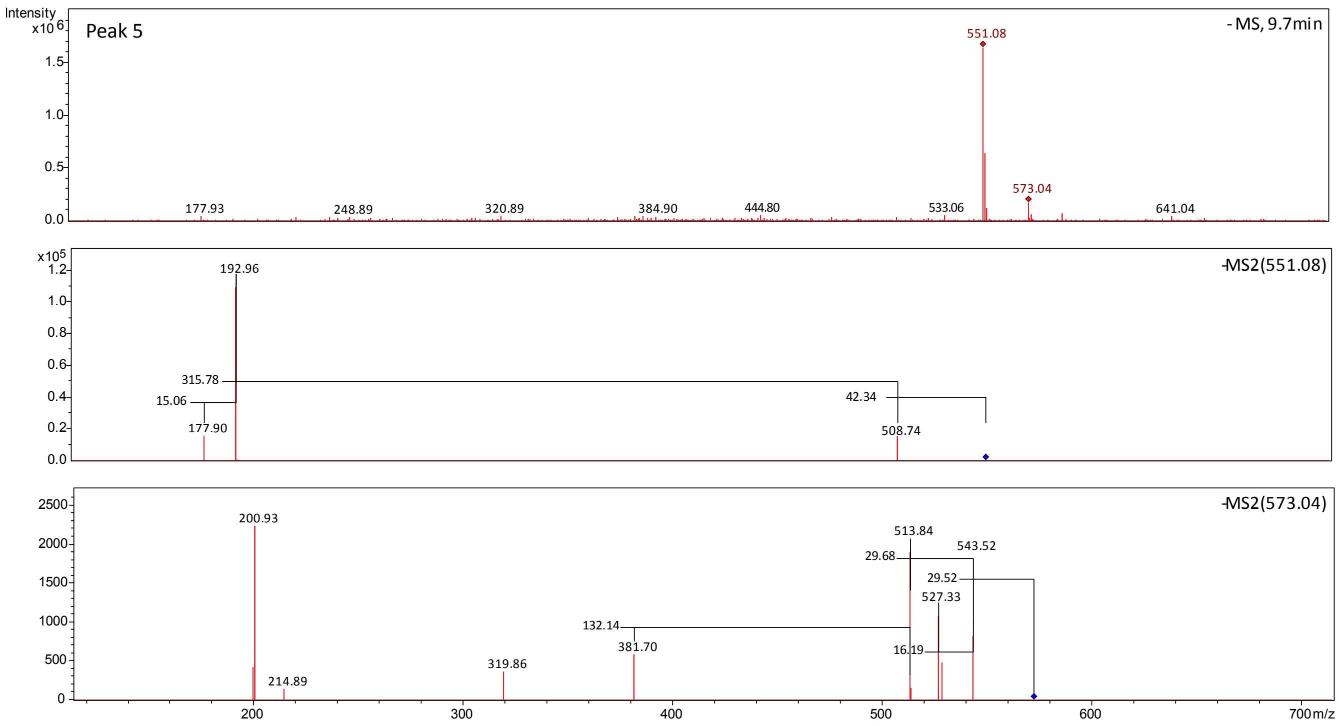
Intensity x10^6
Peak 5
- MS, 9.7min
551.08
573.04
177.93
248.89
320.89
384.90
444.80
533.06
641.04
-MS2(551.08)
192.96
315.78
15.06
177.90
42.34
508.74
-MS2(573.04)
200.93
513.84
543.52
29.68
29.52
527.33
132.14
381.70
16.19
319.86
214.89
m/z

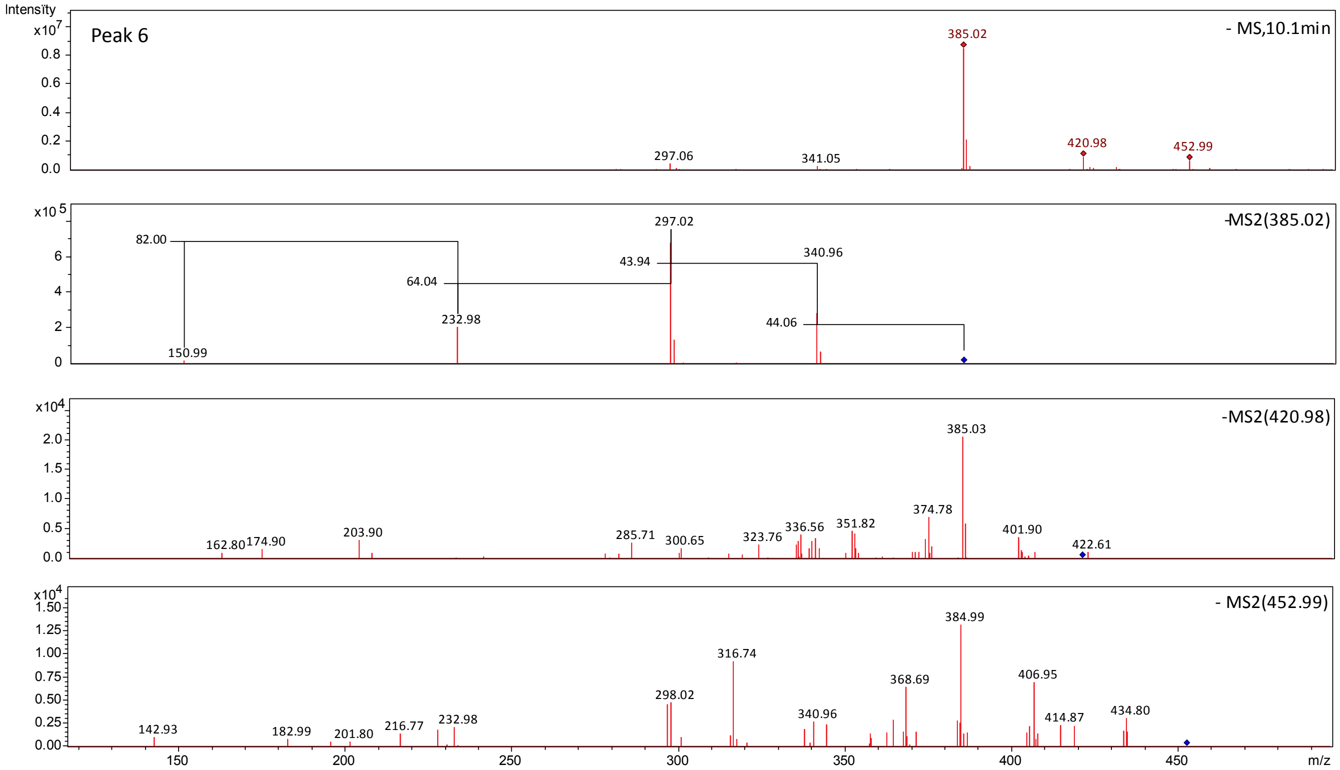

Figure S7. Evolution profile of ferulic acid oxidation by *Ganoderma lucidum* laccase. LC chromatograms at different reaction times are shown: 0 minutes (light blue), 10 minutes (yellow), 25 minutes (violet) and 50 minutes (red). MS and MS/MS spectra for each peak are also shown. All ions are observed as $[M-H]^-$. As reaction time is increasing substrate peak (peak 1) is decreasing in intensity, while product peaks (peaks 2-6) are increasing in intensity, meaning that the substrate is being consumed and the products formed. Major losses during fragmentation are indicated on the MS/MS spectra when relevant. Product peaks are not always characterized by one single mass, but two or more different ones elute at the same time. Whether it is a result of co-elution of two or more different product or chemical degradation has not be investigated. Ferulic acid is identified with the *m/z* 192.99 and a retention time (RT) of 7.6 min. After 10 minutes reaction time four different product peaks are present with RT 9.4, 9.7, 9.9 and 10.3 (peak 3, 4, 5 and 6), while a fifth product peak is appearing after 25 minutes at RT 8.9 min (peak 2). Three different isomers of ferulic acid dimer are present in peak 3, 4 and 6 characterized by *m/z* 385.02. The decarboxylated dimer *m/z* 341.06 (characterized by the loss of 44) is present in two different isomeric forms (peak 2 and 3). Sodium formate adducts of the dimer can be found under peak 3 and 6 characterized by *m/z* 453. Peak 2, 4, 5 and 6 contain masses that could not be immediately resolved to a proposed structure. Peak * RT 11.3 min, containing *m/z* 293.13 and *m/z* 361.13, where the latter is a sodium formate adduct of *m/z* 293.13, was also found by Petrucci *et al.*[1] (corresponding MS spectra is shown in Supplementary Figure S11). It is, most likely, an impurity of LC-MS eluent system since it is present also in the blank run. Suggested chemical structure for the major products are shown in Supplementary Figure S13.

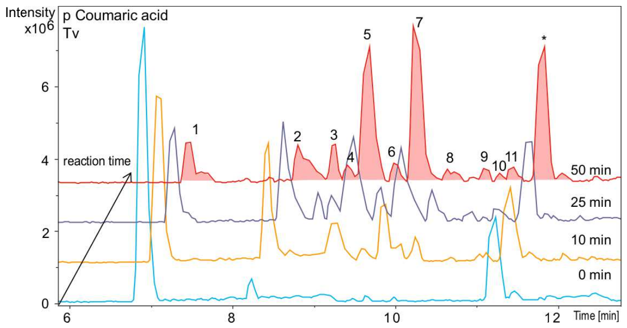
Intensity x10^6
p Coumaric acid
Tv
reaction time
50 min
25 min
10 min
0 min
1 2 3 4 5 6 7 8 9 10 11 *
0 2 4 6
6 8 10 12
Time [min]

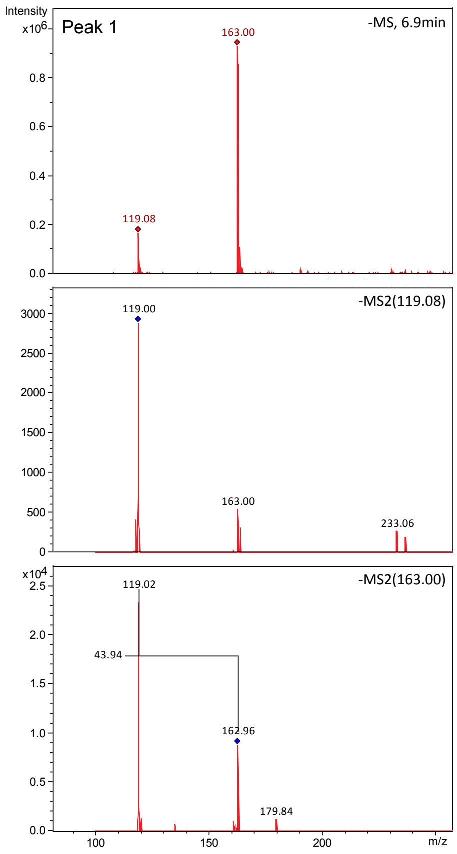
Intensity x10^6
Peak 1
-MS, 6.9min
163.00
119.08
-MS2(119.08)
119.00
163.00
233.06
x10^4
-MS2(163.00)
119.02
43.94
162.96
179.84
100 150 200 m/z

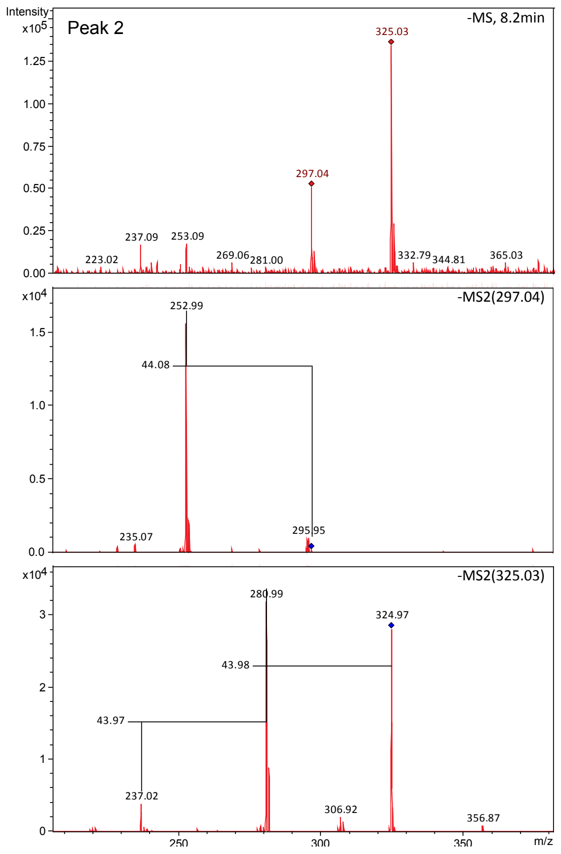
Intensity x10^5
Peak 2
-MS, 8.2min
325.03
297.04
223.02
237.09
253.09
269.06
281.00
332.79
344.81
365.03
x10^4
-MS2(297.04)
252.99
44.08
235.07
295.95
x10^4
-MS2(325.03)
280.99
324.97
43.98
43.97
237.02
306.92
356.87
250 300 350 m/z

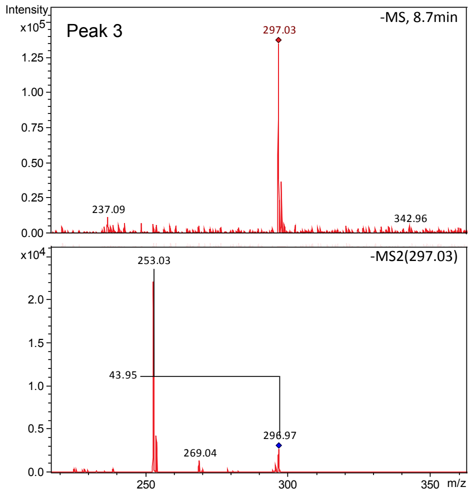
Intensity x10^5
Peak 3
-MS, 8.7min
297.03
237.09
342.96
x10^4
-MS2(297.03)
253.03
43.95
269.04
296.97
250 300 350 m/z

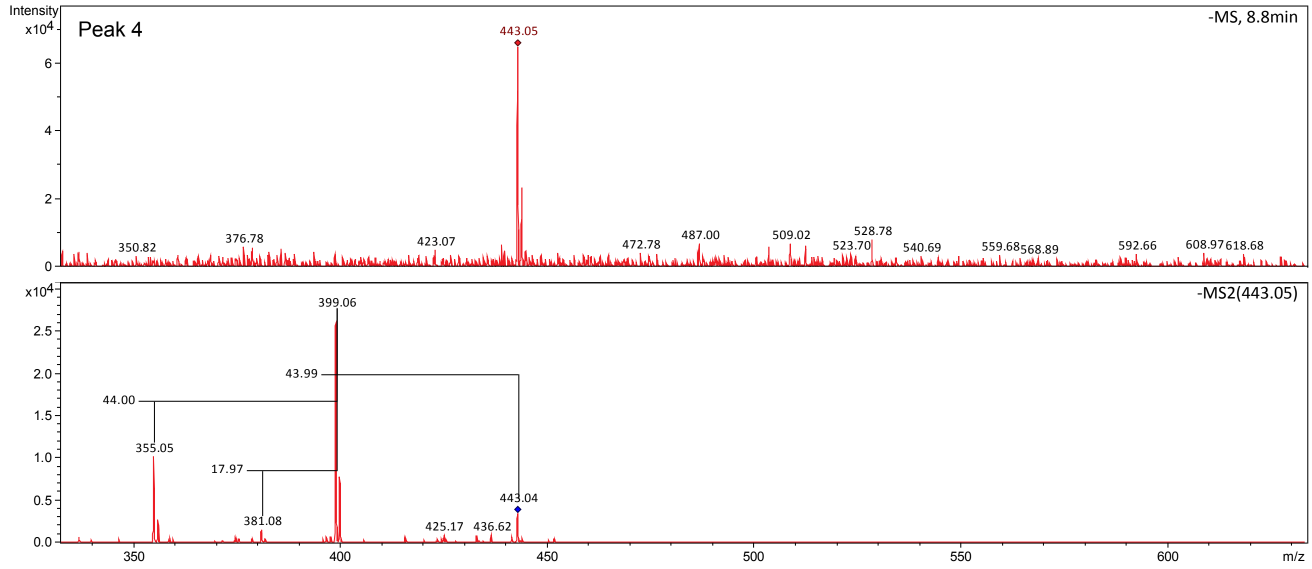
Peak 4
-MS, 8.8min
Intensity
x10^4
443.05
350.82
376.78
423.07
472.78
487.00
509.02
523.70
528.78
540.69
559.68
568.89
592.66
608.97
618.68
-MS2(443.05)
399.06
43.99
44.00
355.05
17.97
381.08
443.04
425.17
436.62
m/z

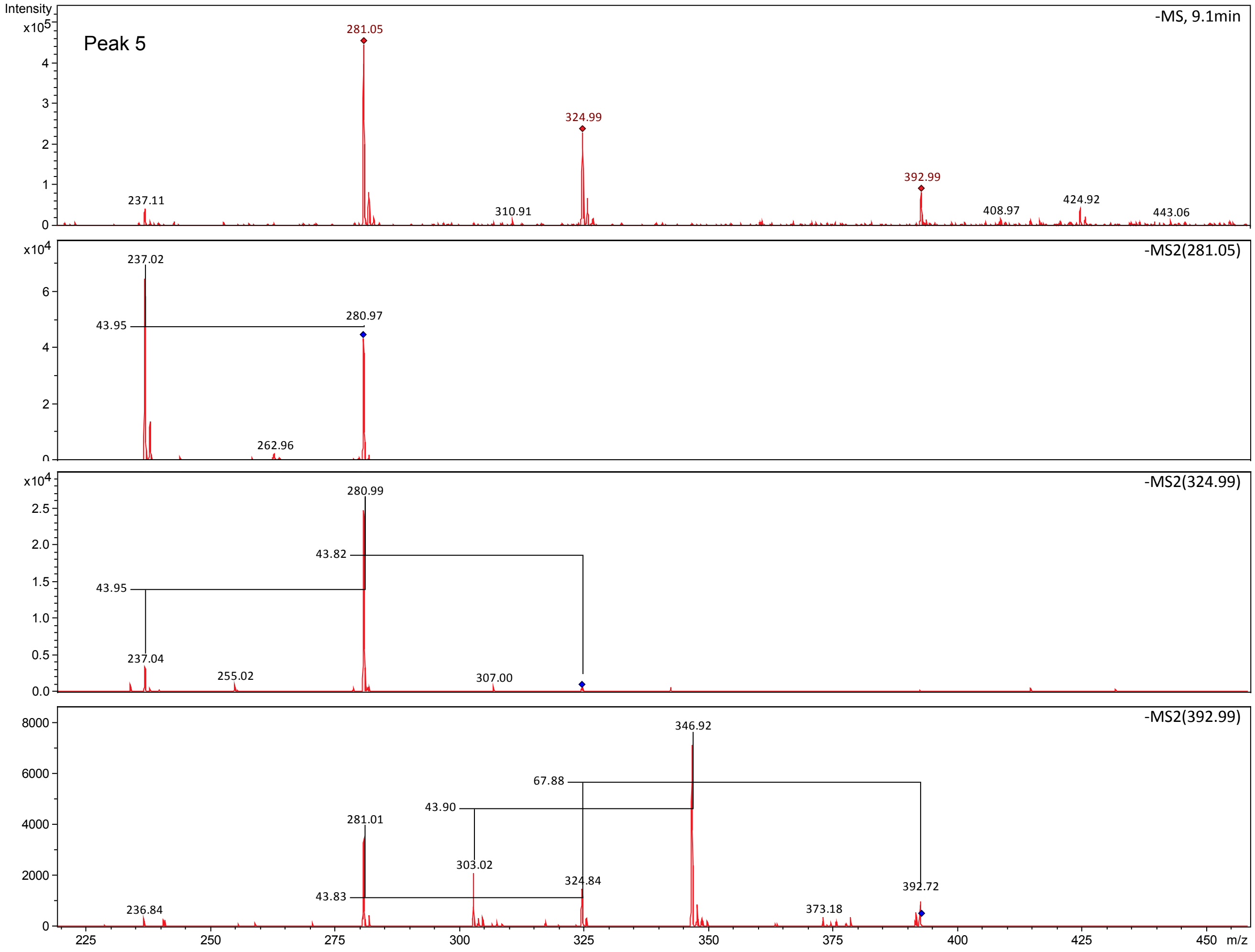
Peak 5
-MS, 9.1min
Intensity
x10^5
281.05
324.99
392.99
237.11
310.91
408.97
424.92
443.06
-MS2(281.05)
237.02
43.95
280.97
262.96
-MS2(324.99)
280.99
43.82
43.95
237.04
255.02
307.00
-MS2(392.99)
346.92
67.88
43.90
281.01
303.02
324.84
43.83
236.84
373.18
392.72
m/z

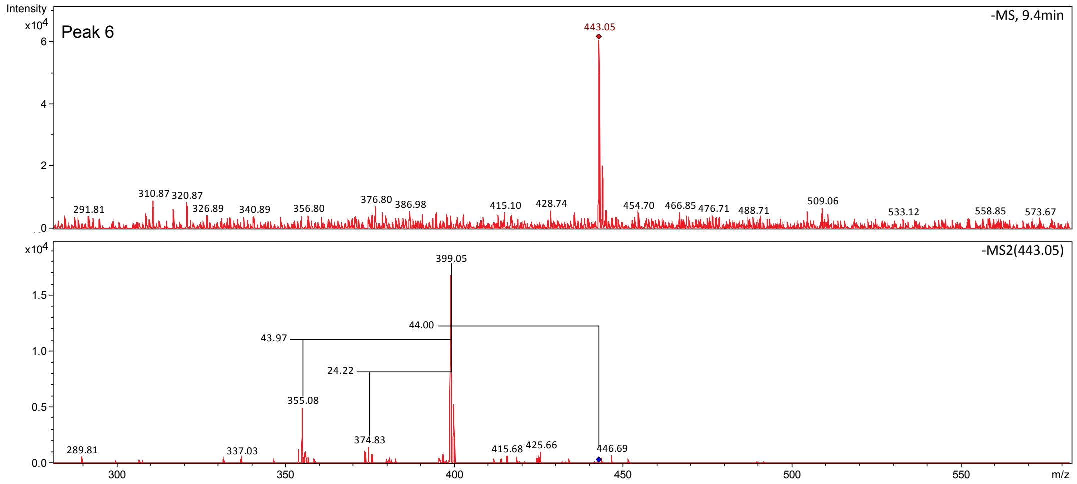
Intensity
x10^4
Peak 6
-MS, 9.4min
443.05
291.81
310.87
320.87
326.89
340.89
356.80
376.80
386.98
415.10
428.74
454.70
466.85
476.71
488.71
509.06
533.12
558.85
573.67
-MS2(443.05)
399.05
44.00
43.97
24.22
355.08
374.83
289.81
337.03
415.68
425.66
446.69
m/z

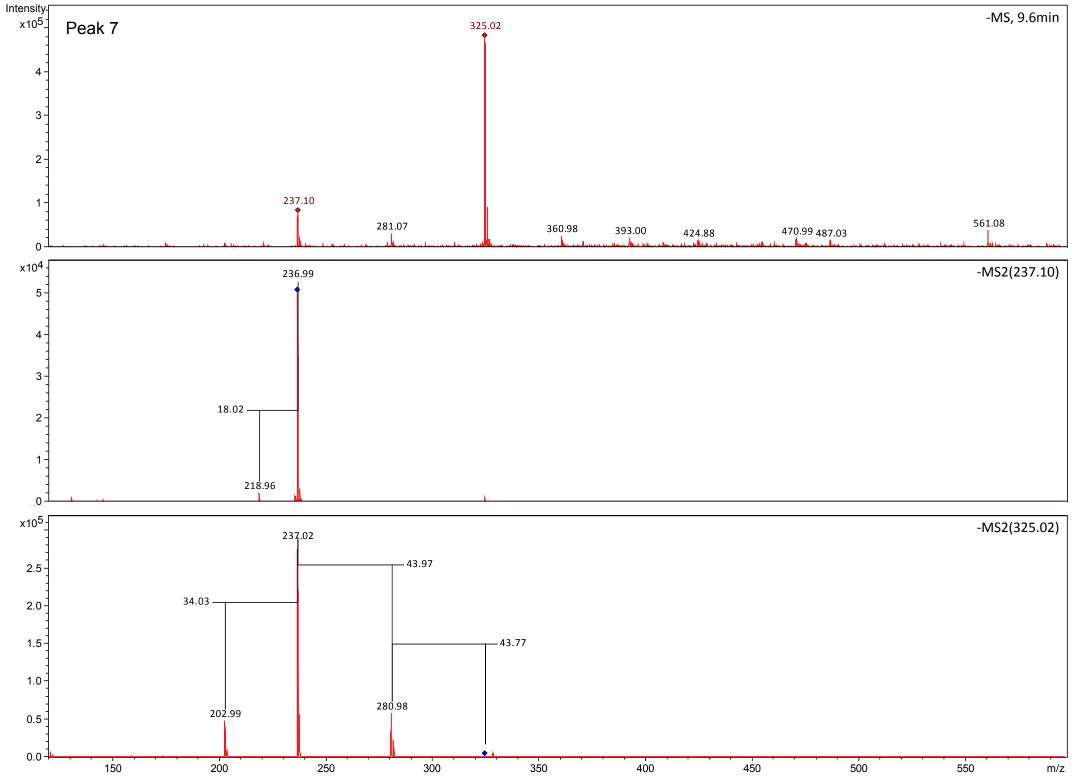
Intensity
x10^5
Peak 7
-MS, 9.6min
325.02
237.10
281.07
360.98
393.00
424.88
470.99
487.03
561.08
-MS2(237.10)
236.99
18.02
218.96
-MS2(325.02)
237.02
43.97
34.03
43.77
202.99
280.98
m/z

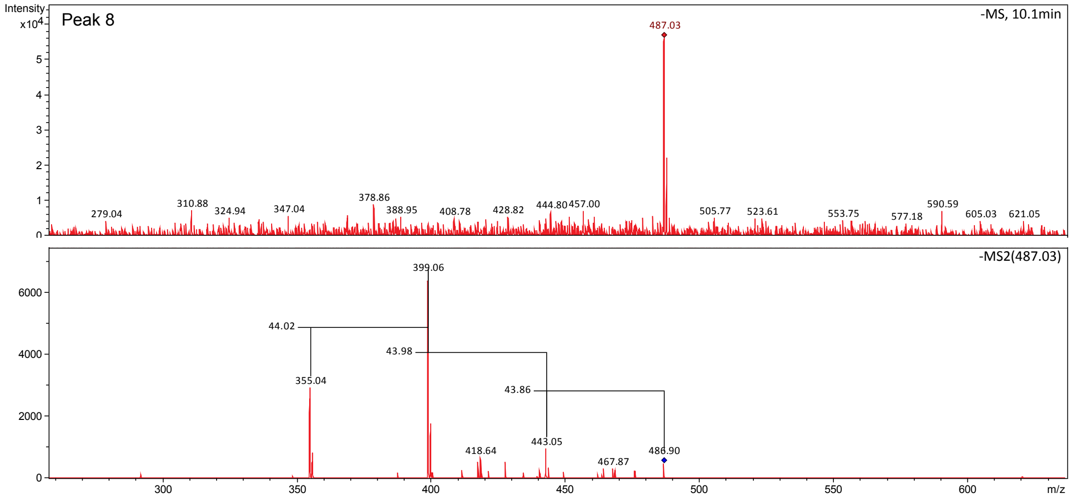
Intensity
x10^4
Peak 8
-MS, 10.1min
487.03
279.04
310.88
324.94
347.04
378.86
388.95
408.78
428.82
444.80
457.00
505.77
523.61
553.75
577.18
590.59
605.03
621.05
-MS2(487.03)
399.06
44.02
43.98
355.04
43.86
418.64
443.05
467.87
486.90
m/z

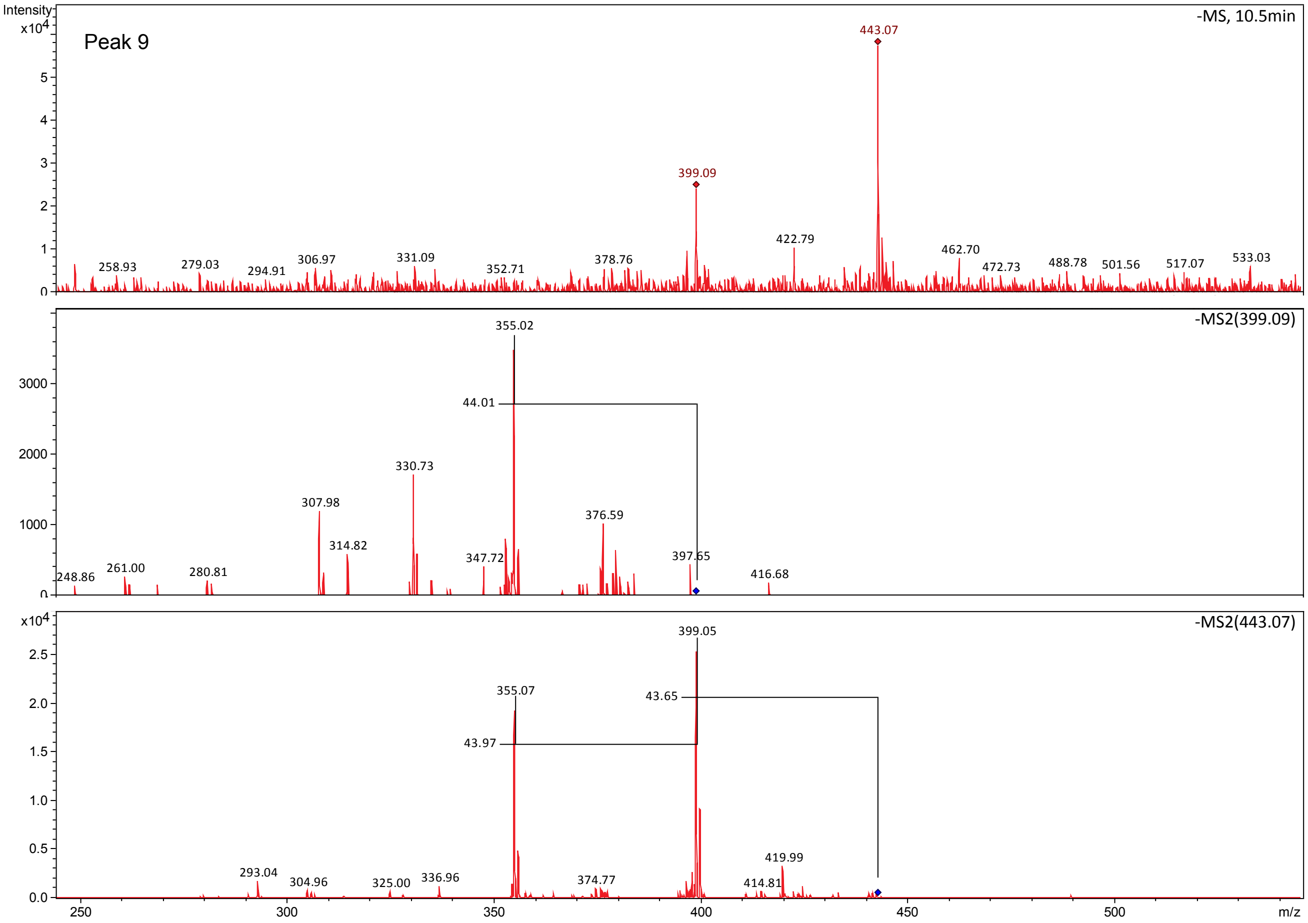
Peak 9
-MS, 10.5min
Intensity
x10^4
443.07
399.09
422.79
258.93
279.03
294.91
306.97
331.09
352.71
378.76
462.70
472.73
488.78
501.56
517.07
533.03
-MS2(399.09)
355.02
44.01
330.73
307.98
314.82
376.59
347.72
397.65
416.68
248.86
261.00
280.81
-MS2(443.07)
x10^4
399.05
355.07
43.65
43.97
293.04
304.96
325.00
336.96
374.77
419.99
414.81
m/z

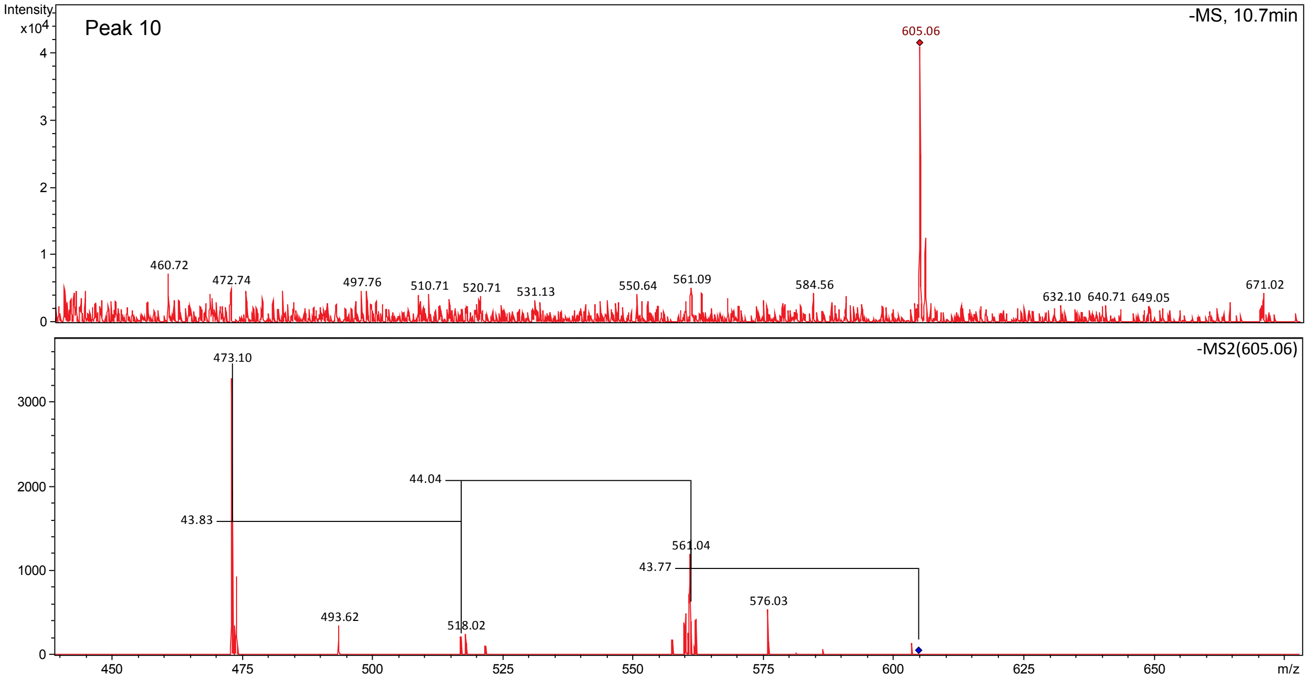
Peak 10
-MS, 10.7min
Intensity
x10^4
605.06
460.72
472.74
497.76
510.71
520.71
531.13
550.64
561.09
584.56
632.10
640.71
649.05
671.02
-MS2(605.06)
473.10
44.04
43.83
561.04
43.77
576.03
493.62
518.02
m/z

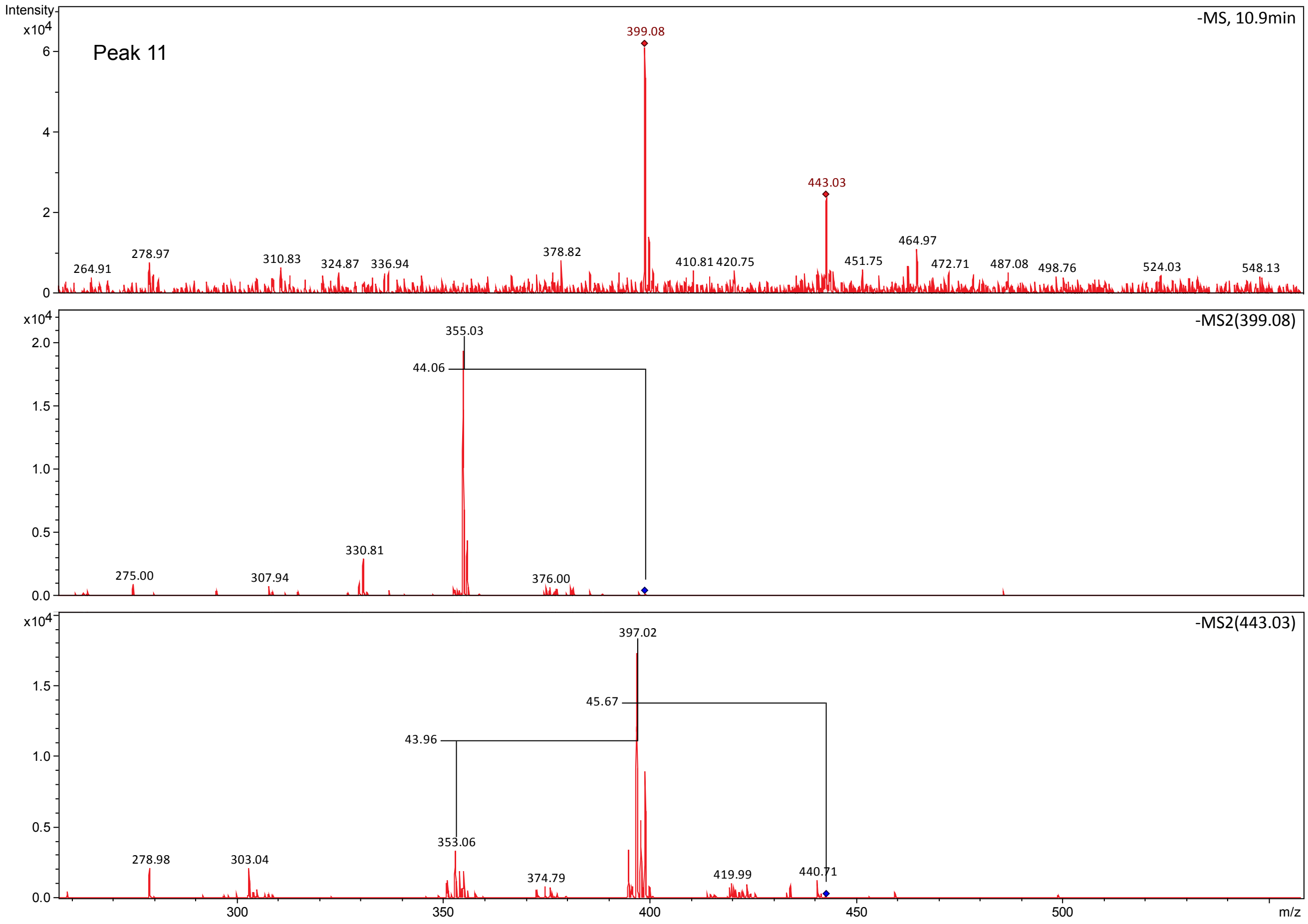

Figure S8. Evolution profile of *p*-coumaric acid oxidation by *Trametes versicolor* laccase. LC chromatograms at different reaction times are shown: 0 minutes (light blue), 10 minutes (yellow), 25 minutes (violet) and 50 minutes (red). MS and MS/MS spectra for each peak are also shown. All ions are observed as $[M\text{-}H]^-$. As reaction time is increasing substrate peak (peak 1) is decreasing in intensity, while product peaks are both increasing and decreasing, meaning that the substrate is being consumed and the products formed and then degraded or subjected to further modifications. Major losses during fragmentation are indicated on the MS/MS spectra when relevant. Product peaks are not always characterized by one single mass, but two or more different compounds elute at the same time. Whether it is a result of co-elution of two or more different product or chemical degradation has not be investigated. *p*-coumaric acid (peak 1) is identified with the *m/z* 163.00 and a retention time (RT) of 6.9 min. The product profile is complex compared to the ones from sinapic acid and ferulic acid ones. Three different dimer isomers *m/z* 325.03 are formed already after 10 minutes of reaction at RT 8.3, 9.2 and 9.7 min (peak 2, 5 and 7). Peak 2 also contains the mass of the decarboxylated dimer *m/z* 281.05 (loss of 44) and a sodium formate adduct (+ 68) of the dimer *m/z* 393. After 10 minutes also the trimer *m/z* 487.03 is present RT 10.1 min (peak 8). At longer reaction times (equal or longer than 25 minutes) four isomers of the decarboxylated trimer *m/z* 443.03 are found in peak 4, 6, 9 and 11 as well as the double decarboxylated trimer *m/z* 399.08 in peak 9 and 11. Peak 10 is characterized by *m/z* 605.06 which corresponds to the mass of the decarboxylated tetramer. Peak 2 and 3 contain masses that could not be immediately resolved to a proposed structure. Peak * RT 11.3 min, containing *m/z* 293.13 and *m/z* 361.13, where the latter is a sodium formate adduct of *m/z* 293.13, was also found by Petrucci *et al.*[1] (corresponding MS spectra is shown in Supplementary Figure S10). It is, most likely, an impurity of LC-MS eluent system since it is present also in the blank run. Suggested chemical structure for the major products are shown in Supplementary Figure S14.

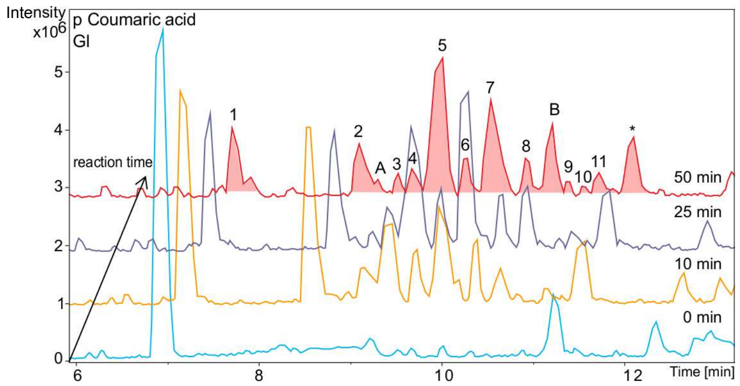
Intensity x10^6
p Coumaric acid
GI
reaction time
50 min
25 min
10 min
0 min
Time [min]
1 2 A 3 4 5 6 7 8 B 9 10 11 *

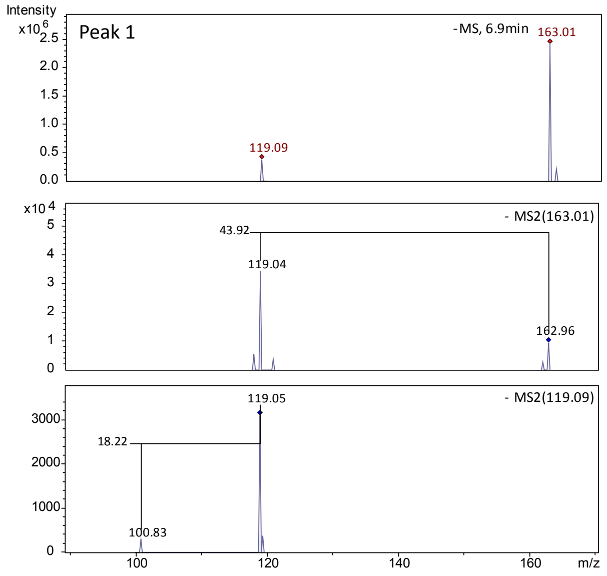
Peak 1
-MS, 6.9min
163.01
119.09
- MS2(163.01)
43.92
119.04
162.96
- MS2(119.09)
119.05
18.22
100.83
m/z

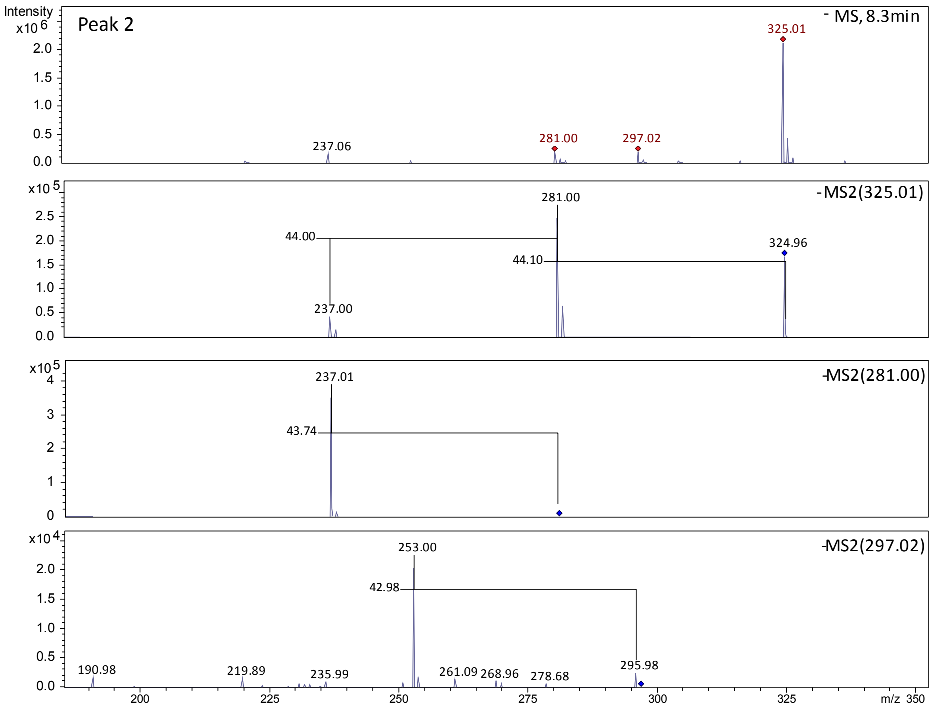
Peak 2
- MS, 8.3min
325.01
237.06
281.00
297.02
-MS2(325.01)
281.00
44.00
44.10
324.96
237.00
-MS2(281.00)
237.01
43.74
-MS2(297.02)
253.00
42.98
190.98
219.89
235.99
261.09
268.96
278.68
295.98
m/z

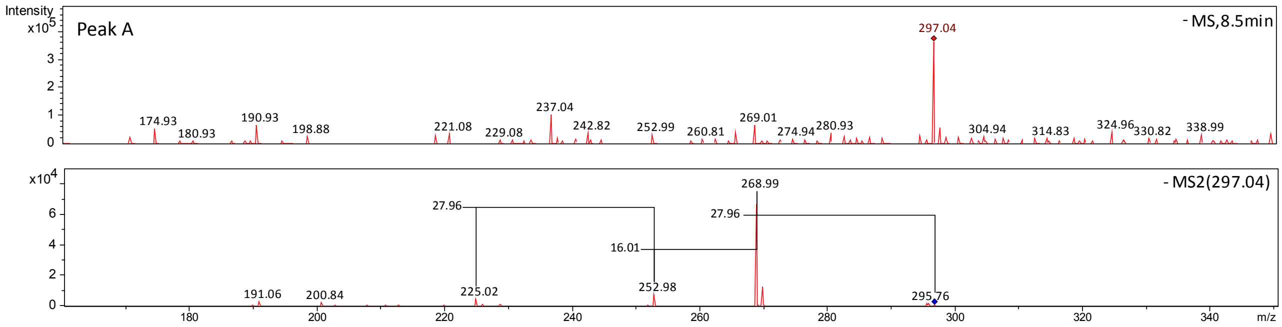
Peak A
- MS,8.5min
Intensity x10^5
297.04
174.93 180.93 190.93 198.88 221.08 229.08 237.04 242.82 252.99 260.81 269.01 274.94 280.93 304.94 314.83 324.96 330.82 338.99
- MS2(297.04)
x10^4
268.99
27.96
16.01
191.06 200.84 225.02 252.98 295.76
m/z

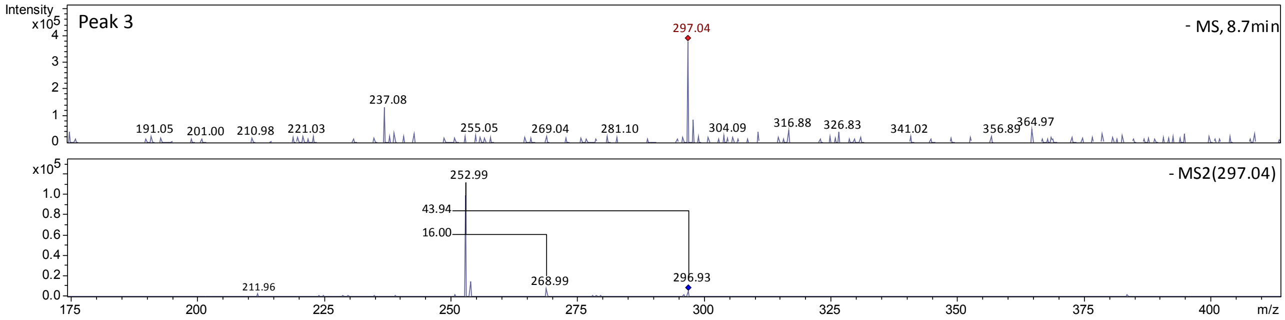
Peak 3
- MS, 8.7min
Intensity x10^5
297.04
191.05 201.00 210.98 221.03 237.08 255.05 269.04 281.10 304.09 316.88 326.83 341.02 356.89 364.97
- MS2(297.04)
x10^5
252.99
43.94
16.00
211.96 268.99 296.93
m/z

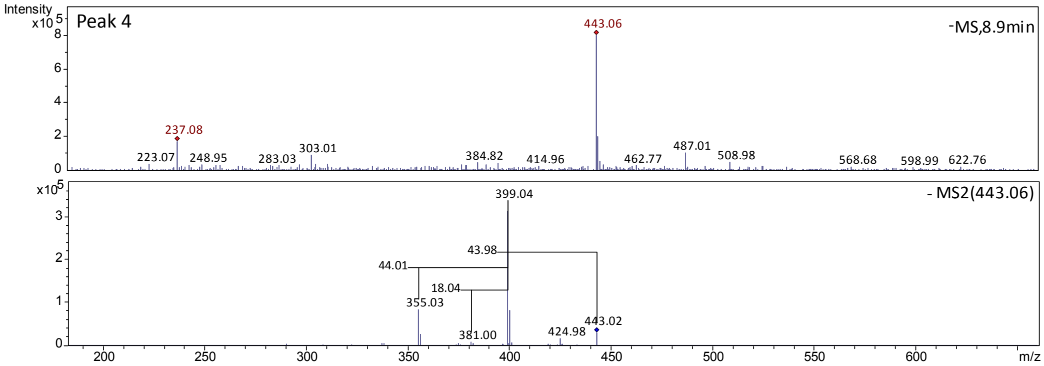
Peak 4
-MS,8.9min
Intensity x10^5
443.06
237.08
223.07 248.95 283.03 303.01 384.82 414.96 462.77 487.01 508.98 568.68 598.99 622.76
- MS2(443.06)
x10^5
399.04
43.98
44.01
18.04
355.03 381.00 424.98 443.02
m/z

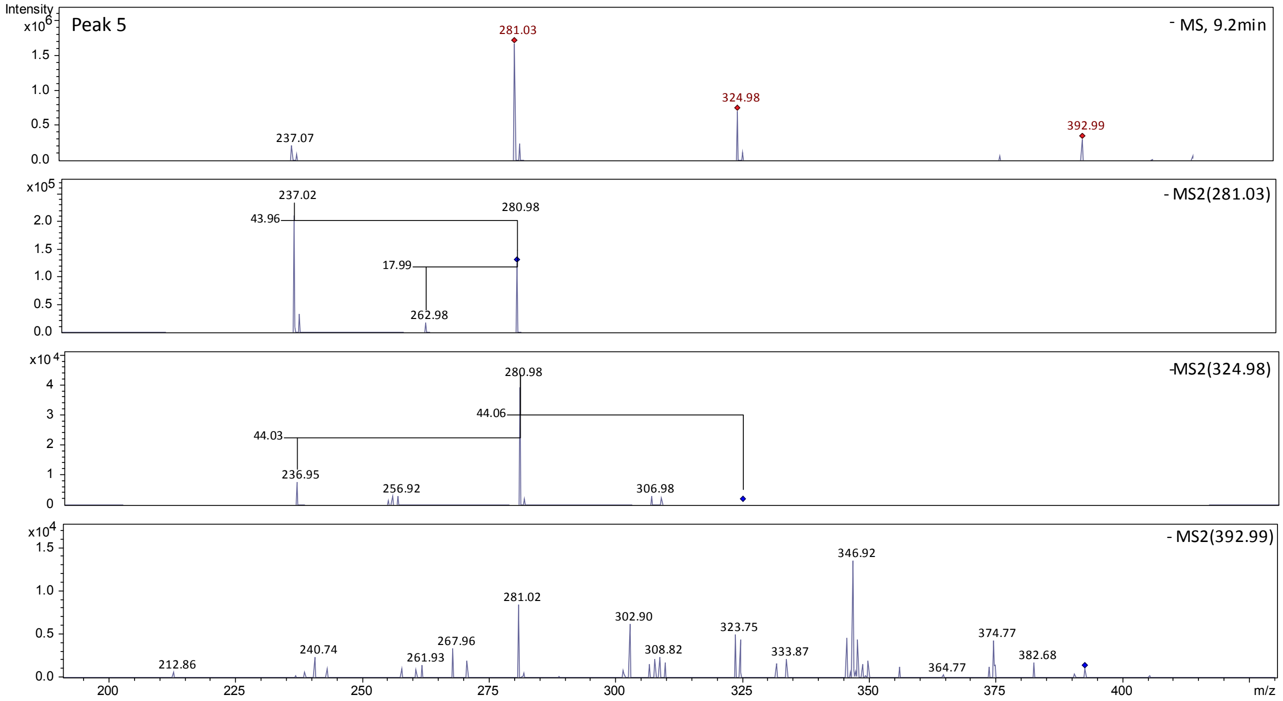
Peak 5
- MS, 9.2min
Intensity x10^6
281.03 324.98 392.99 237.07
- MS2(281.03)
x10^5
237.02 280.98
43.96
17.99
262.98
-MS2(324.98)
x10^4
280.98
44.06
44.03
236.95 256.92 306.98
- MS2(392.99)
x10^4
346.92 281.02 302.90 323.75 308.82 374.77 382.68 333.87 212.86 240.74 261.93 267.96 364.77
m/z

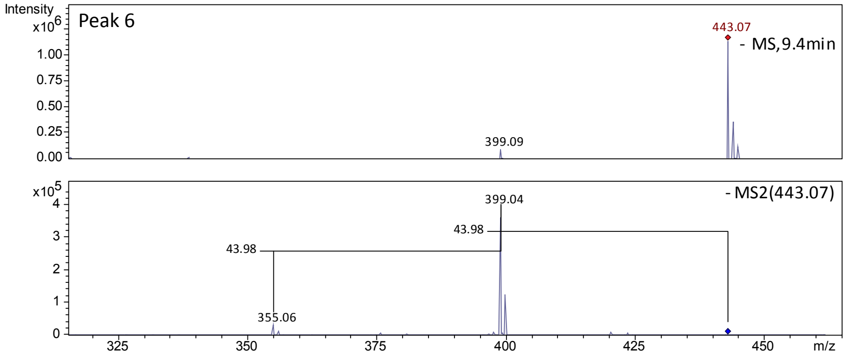
Intensity
x10^6
Peak 6
443.07
- MS,9.4min
399.09
x10^5
- MS2(443.07)
399.04
43.98
43.98
355.06
m/z

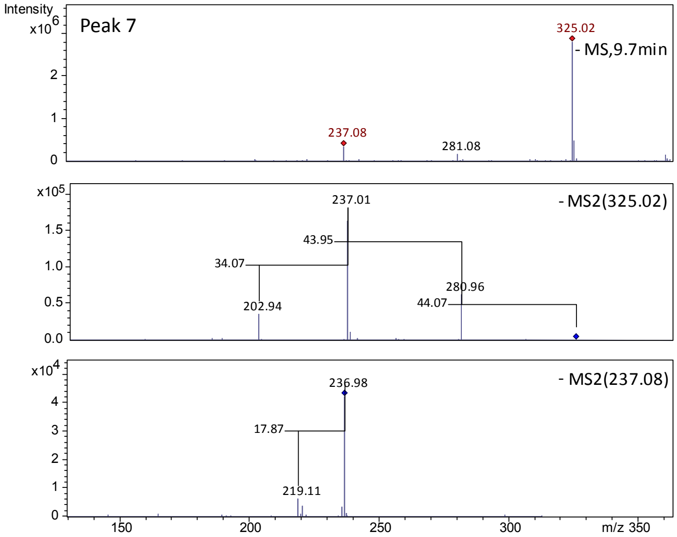
Intensity
x10^6
Peak 7
325.02
- MS,9.7min
237.08
281.08
x10^5
- MS2(325.02)
237.01
43.95
34.07
280.96
44.07
202.94
x10^4
- MS2(237.08)
236.98
17.87
219.11
m/z

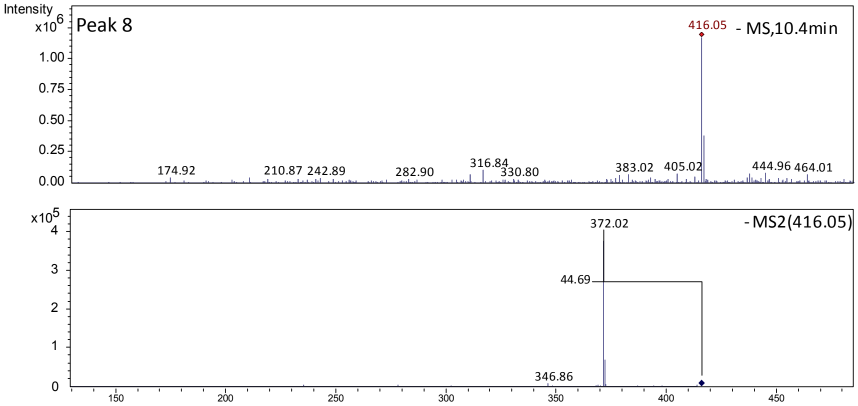
Intensity
x10^6
Peak 8
416.05
- MS,10.4min
174.92
210.87
242.89
282.90
316.84
330.80
383.02
405.02
444.96
464.01
x10^5
- MS2(416.05)
372.02
44.69
346.86

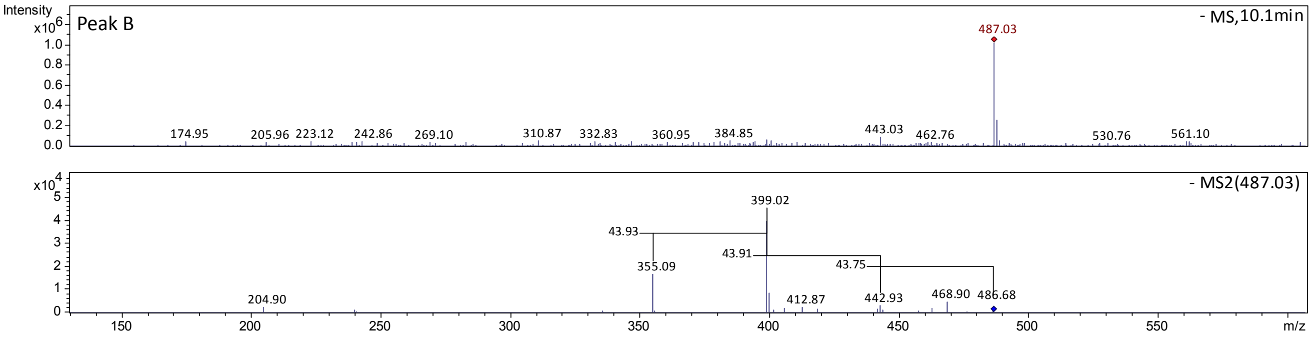
Intensity
x10^6
Peak B
- MS,10.1min
487.03
174.95
205.96
223.12
242.86
269.10
310.87
332.83
360.95
384.85
443.03
462.76
530.76
561.10
x10^4
- MS2(487.03)
399.02
43.93
43.91
43.75
355.09
204.90
412.87
442.93
468.90
486.68
m/z

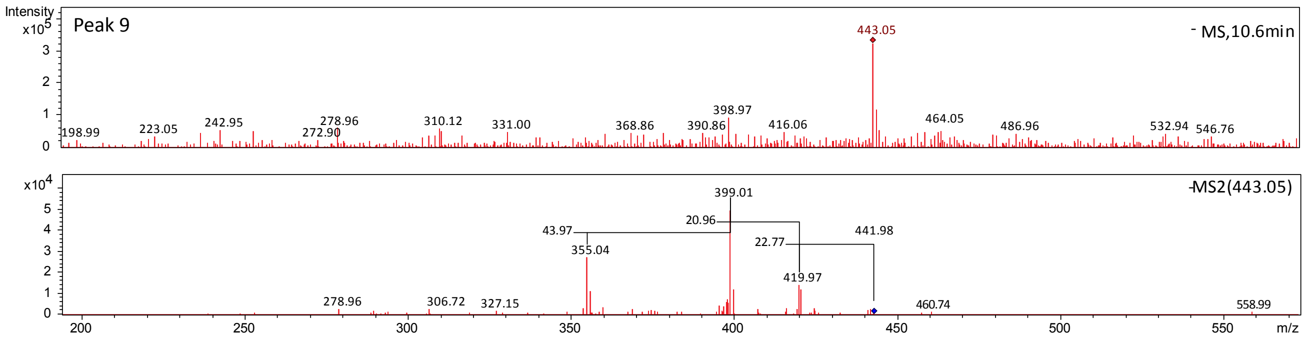
Intensity
x10^5
Peak 9
- MS,10.6min
443.05
198.99
223.05
242.95
272.90
278.96
310.12
331.00
368.86
390.86
398.97
416.06
464.05
486.96
532.94
546.76
x10^4
-MS2(443.05)
399.01
20.96
43.97
355.04
22.77
441.98
419.97
278.96
306.72
327.15
460.74
558.99
m/z

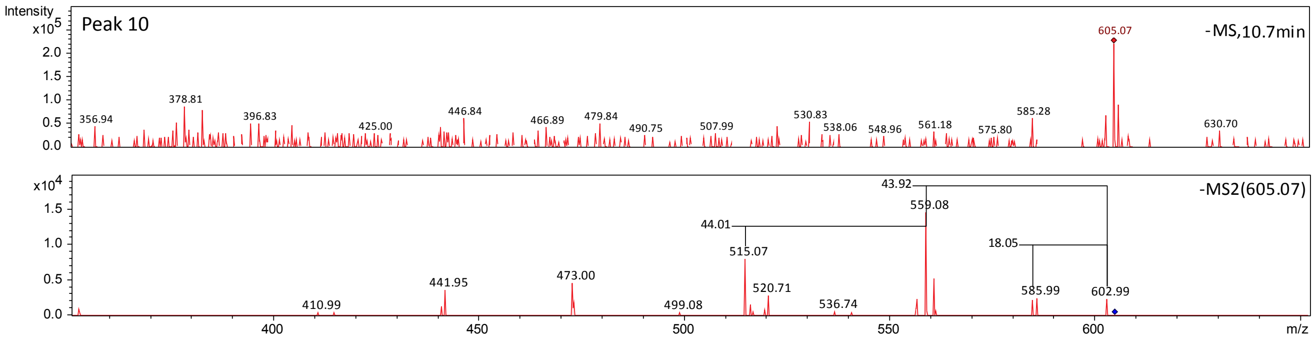
Intensity
x10^5
Peak 10
- MS,10.7min
605.07
356.94
378.81
396.83
425.00
446.84
466.89
479.84
490.75
507.99
530.83
538.06
548.96
561.18
575.80
585.28
630.70
x10^4
-MS2(605.07)
43.92
559.08
44.01
515.07
18.05
441.95
473.00
410.99
499.08
520.71
536.74
585.99
602.99
m/z

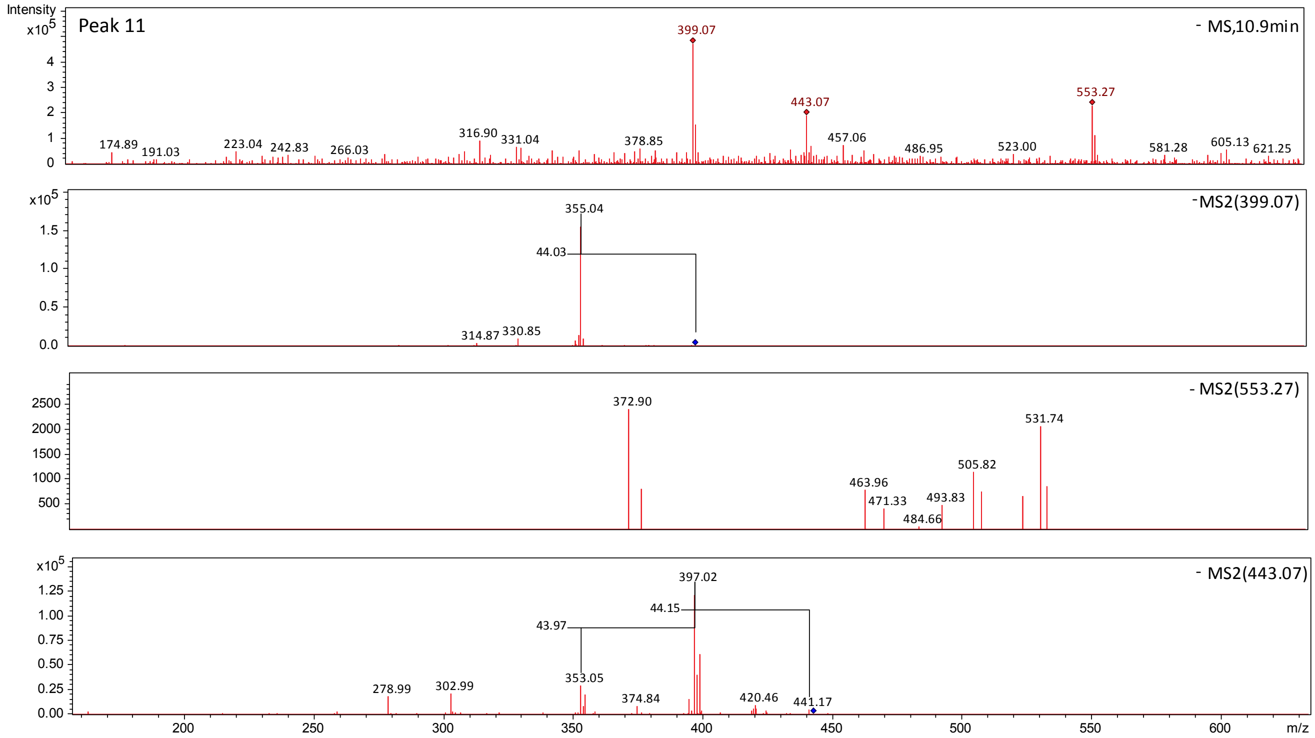
Intensity
x10^5
Peak 11
- MS,10.9min
399.07
443.07
553.27
174.89
191.03
223.04
242.83
266.03
316.90
331.04
378.85
457.06
486.95
523.00
581.28
605.13
621.25
x10^5
-MS2(399.07)
355.04
44.03
314.87
330.85
- MS2(553.27)
372.90
531.74
505.82
463.96
471.33
493.83
484.66
x10^5
- MS2(443.07)
397.02
44.15
43.97
353.05
278.99
302.99
374.84
420.46
441.17
m/z

Figure S9. Evolution profile of *p*-coumaric acid oxidation by *Ganoderma lucidum* laccase. LC chromatograms at different reaction times are shown: 0 minutes (light blue), 10 minutes (yellow), 25 minutes (violet) and 50 minutes (red). MS and MS/MS spectra for each peak are also shown. All ions are observed as $[M-H]^-$. As reaction time is increasing substrate peak (peak 1) is decreasing in intensity, while product peaks are both increasing and decreasing, meaning that the substrate is being consumed and the products formed and then degraded or subjected to further modifications. Major losses during fragmentation are indicated on the MS/MS spectra when relevant. Product peaks are not always characterized by one single mass, but two or more different compounds elute at the same time. Whether it is a result of co-elution of two or more different product or chemical degradation has not be investigated. *p*-coumaric acid (peak 1) is identified with the *m/z* 163.00 and a retention time (RT) of 6.9 min. The product profile is complex compared to the ones from sinapic acid and ferulic acid ones. Three different dimer isomers *m/z* 325.03 are formed already after 10 minutes of reaction peak 2, 5 and 7 (RT 8.3, 9.2 and 9.7 min). Peaks 2 and 5 contain also the mass of the decarboxylated dimer *m/z* 281.05 (loss of 44) and a sodium formate adduct (+ 68) of the dimer *m/z* 393 (only in peak 5). A double decarboxylated dimer *m/z* 237.08 (peak 7) is also present, it is not present in the oxidation of *p*-coumaric acid by *Trametes versicolor* laccase. After 10 minutes also the trimer *m/z* 487.03 is present RT 10.1 min (peak 8). At longer reaction times (equal or longer than 25 minutes) four isomers of the decarboxylated trimer *m/z* 443.03 are found in peak 4, 6, 9 and 11 as well as the double decarboxylated trimer *m/z* 399.08 in peak 9 and 11. Peak 10 is characterized by *m/z* 605.06 which corresponds to the mass of the decarboxylated tetramer. Peak 2, A, 3, B and 12 contain masses that could not be immediately resolved to a proposed structure. Peak * RT 11.3 min, containing *m/z* 293.13 and *m/z* 361.13, where the latter is a sodium formate adduct of *m/z* 293.13, was also found by Petrucci *et al.*[1] (corresponding MS spectra is shown in Supplementary Figure S11). It is, most likely, an impurity of LC-MS eluent system since it is present also in the blank run. Suggested chemical structure for the major products are shown in Supplementary Figure S14.

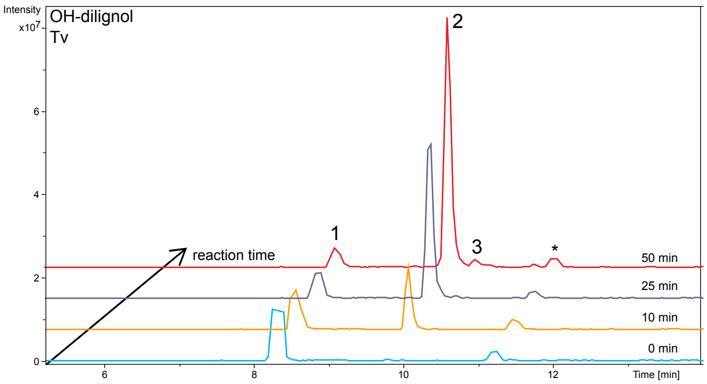
Intensity
x10^7
OH-dilignol
Tv
reaction time
1
2
3
*
50 min
25 min
10 min
0 min
Time [min]

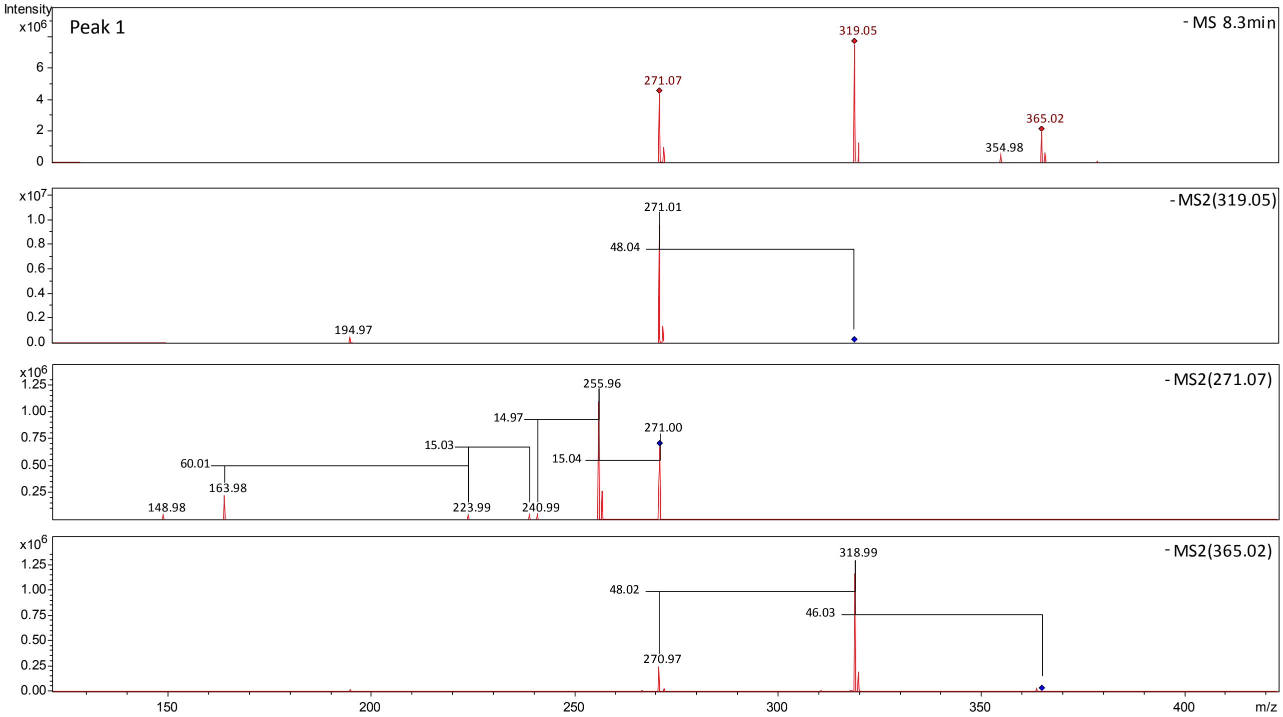
Intensity
x10^6
Peak 1
- MS 8.3min
271.07
319.05
365.02
354.98
- MS2(319.05)
271.01
48.04
194.97
- MS2(271.07)
255.96
14.97
15.03
60.01
15.04
271.00
163.98
148.98
223.99
240.99
- MS2(365.02)
318.99
48.02
46.03
270.97
m/z

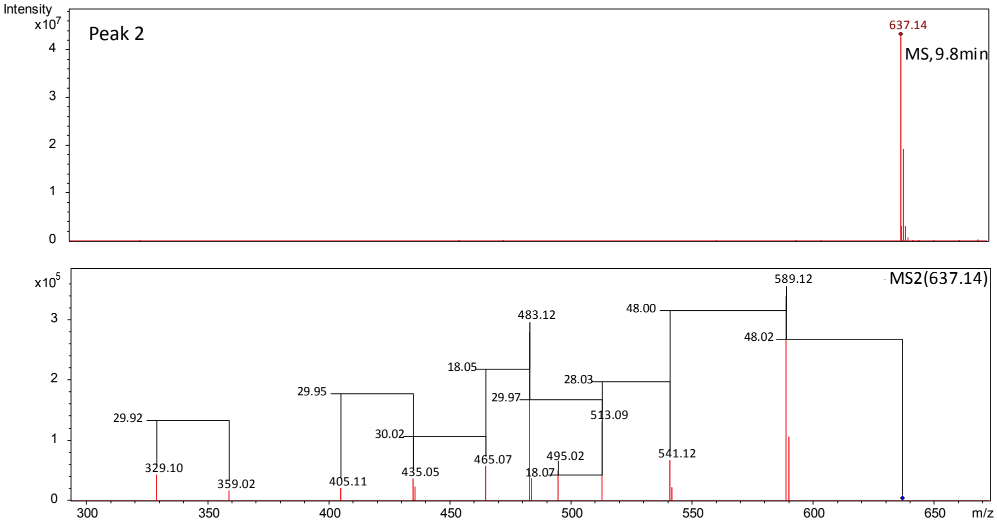
Intensity
x10^7
Peak 2
637.14
MS,9.8min
MS2(637.14)
589.12
483.12
48.00
48.02
18.05
28.03
29.95
29.97
29.92
30.02
513.09
329.10
359.02
405.11
435.05
465.07
495.02
18.07
541.12
m/z

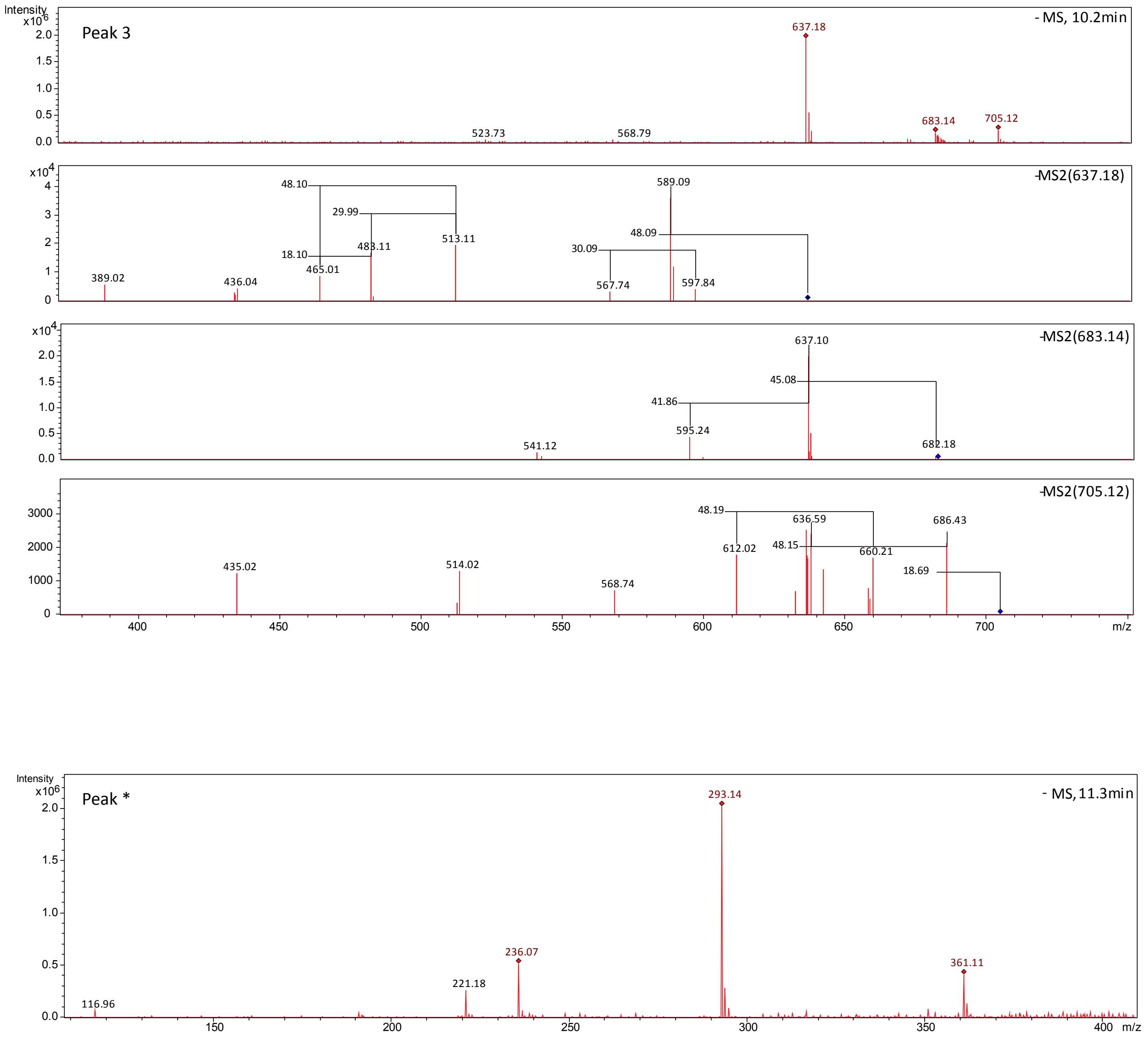

Figure S10. Evolution profile of OH-dilignol oxidation by *Trametes versicolor* laccase. LC chromatograms at different reaction times are shown: 0 minutes (light blue), 10 minutes (yellow), 25 minutes (violet) and 50 minutes (red). MS and MS/MS spectra for each peak are also shown. All ions are observed as $[M\text{-}H]^-$. As reaction time is increasing substrate peak (peak 1) is decreasing in intensity, while product peaks are increasing and decreasing, meaning that the substrate is being consumed and the products formed. Major losses during fragmentation are indicated on the MS/MS spectra when relevant. Product peaks are not always characterized by one single mass, but two or more different compound elute at the same time. Whether it is a result of co-elution of two or more different product or chemical degradation has not be investigated. OH-dilignol (peak 1) is identified with the *m/z* 319.05 and a retention time (RT) of 8.3 min. The product profile is simple, only two isomers of the OH-dilignol dimer *m/z* 637.14 are formed (peak 2 and 3) already after 10 minutes of reaction. A formate adduct (+ 46) of the dimer *m/z* 683.14 and a sodium formate adduct (+ 68) of the dimer *m/z* 705.12 are both present in peak 3. Peak * RT 11.3 min, containing *m/z* 293.13 and *m/z* 361.13, where the latter is a sodium formate adduct of *m/z* 293.13, was also found by Petrucci *et al.*[1]. It is, most likely, an impurity of LC-MS eluent system since it is present also in the blank run. Suggested chemical structure for the major products are shown in Supplementary Figure S15.

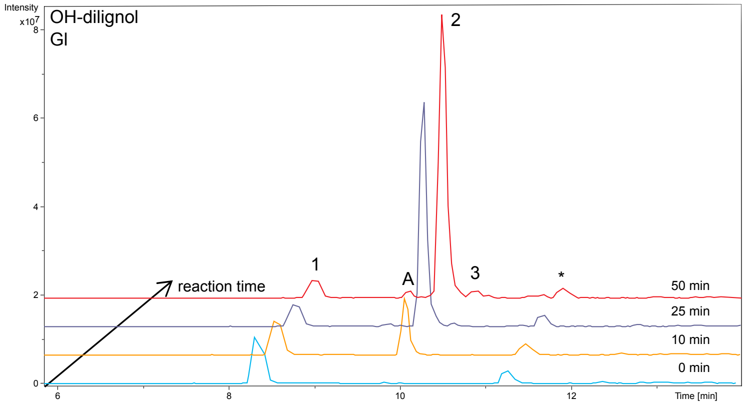
OH-dilignol
GI
1
2
A
3
*
reaction time
50 min
25 min
10 min
0 min
Intensity
x10^7
Time [min]

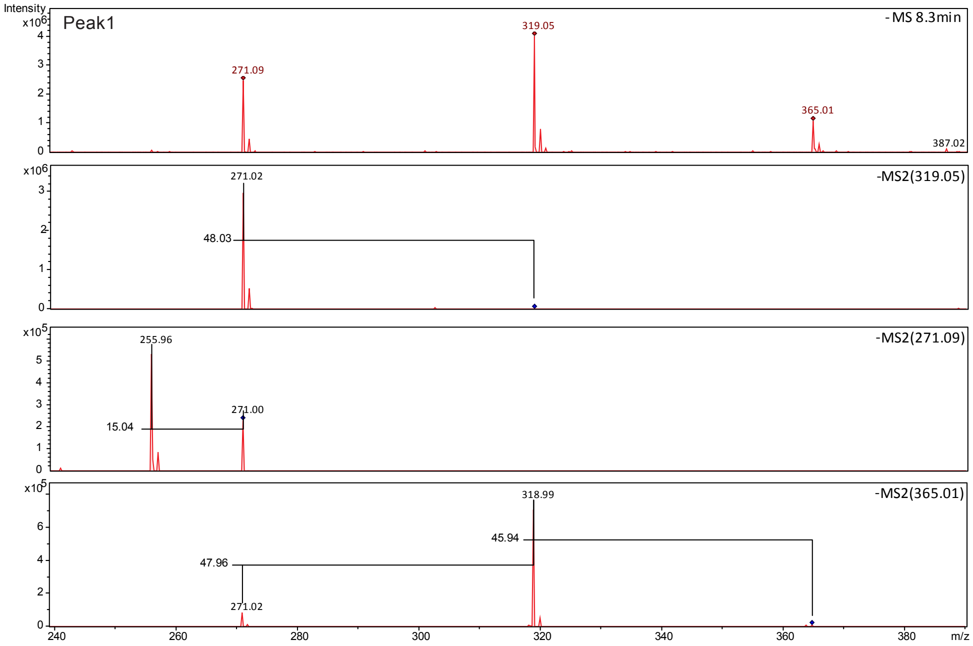
Peak1
-MS 8.3min
271.09
319.05
365.01
387.02
-MS2(319.05)
271.02
48.03
-MS2(271.09)
255.96
271.00
15.04
-MS2(365.01)
318.99
45.94
47.96
271.02
m/z

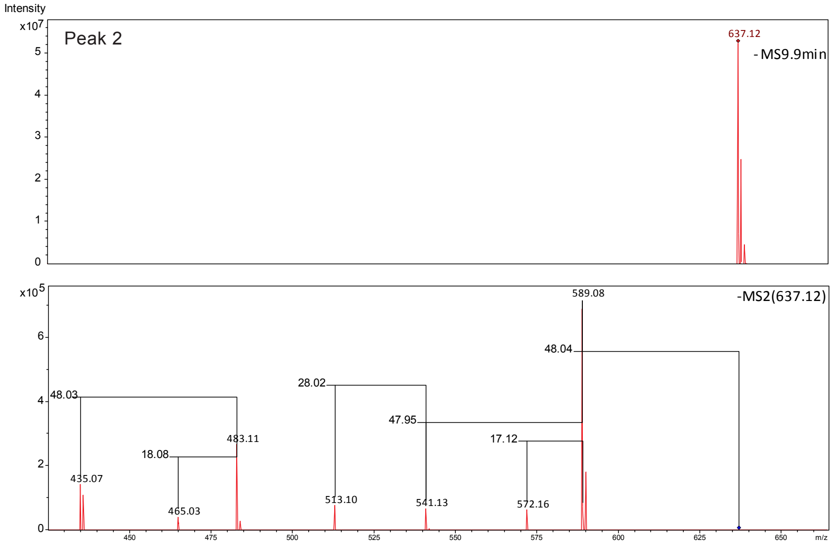
Peak 2
637.12
-MS9.9min
-MS2(637.12)
589.08
48.04
28.02
48.03
47.95
483.11
18.08
17.12
435.07
465.03
513.10
541.13
572.16
m/z

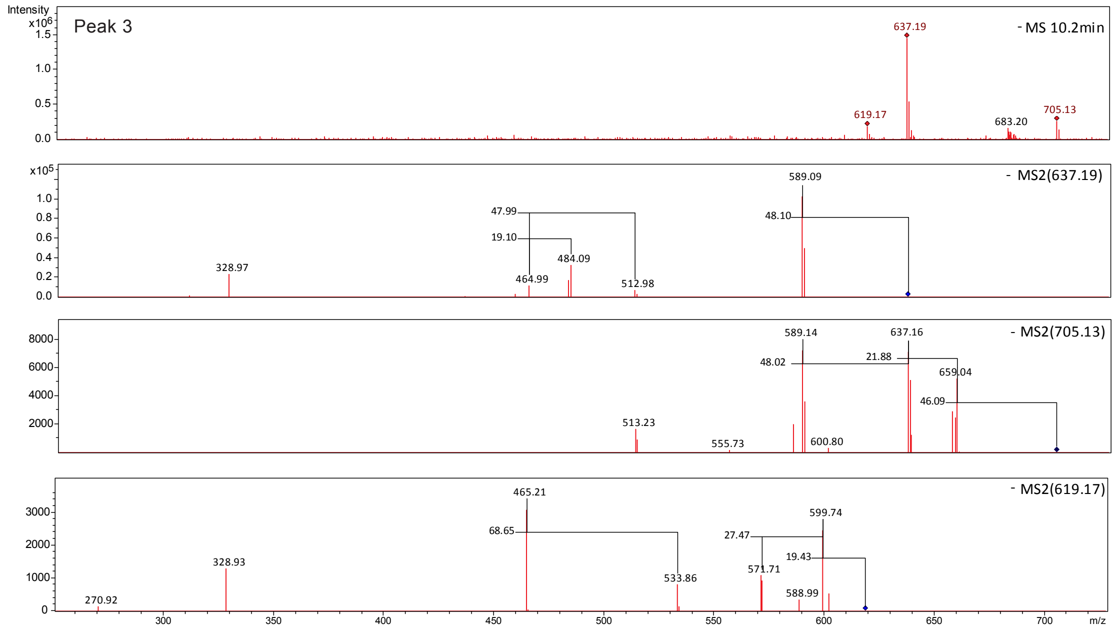
Intensity
x10^6
Peak 3
- MS 10.2min
637.19
619.17
683.20
705.13
x10^5
- MS2(637.19)
589.09
47.99
48.10
19.10
484.09
328.97
464.99
512.98
- MS2(705.13)
589.14
637.16
48.02
21.88
659.04
46.09
513.23
555.73
600.80
- MS2(619.17)
465.21
599.74
68.65
27.47
19.43
328.93
571.71
533.86
588.99
270.92
m/z

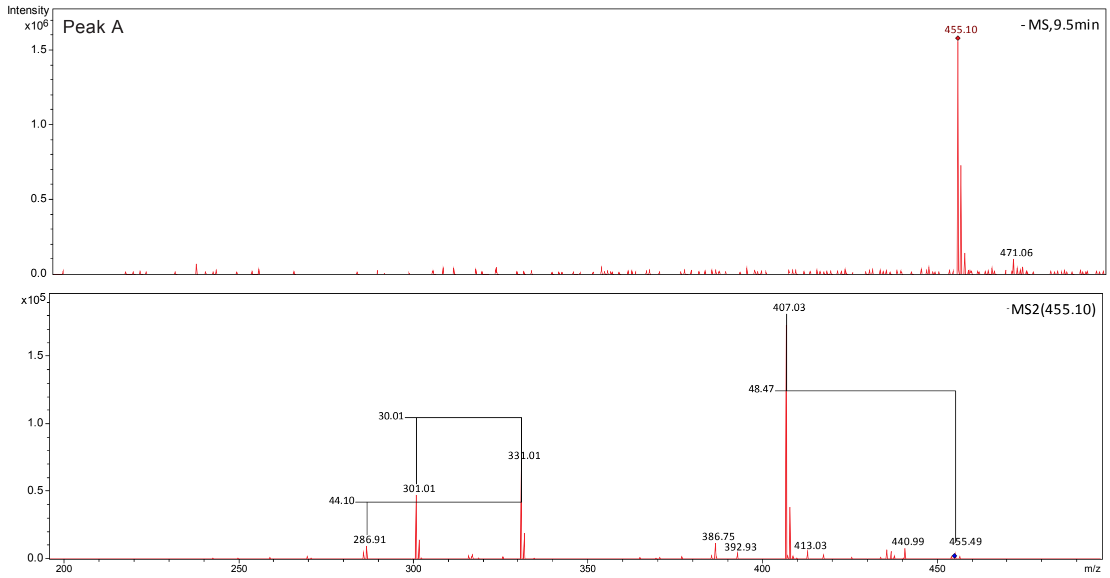
Intensity
x10^6
Peak A
- MS,9.5min
455.10
471.06
x10^5
-MS2(455.10)
407.03
48.47
30.01
331.01
301.01
44.10
286.91
386.75
392.93
413.03
440.99
455.49
m/z

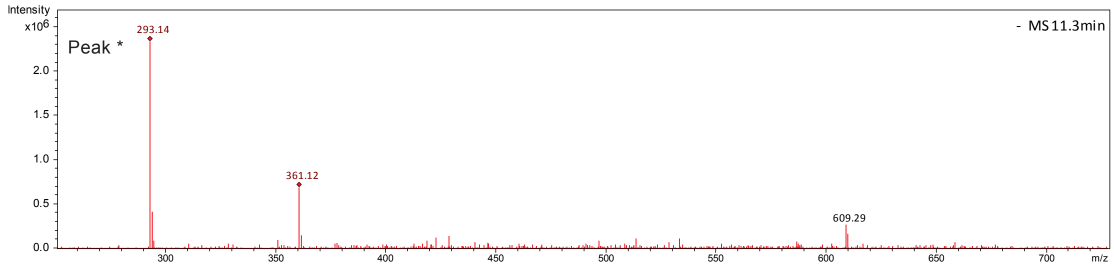
Intensity
x10^6
Peak *
- MS 11.3min
293.14
361.12
609.29
m/z

Figure S11. Evolution profile of OH-dilignol oxidation by *Trametes versicolor* laccase. LC chromatograms at different reaction times are shown: 0 minutes (light blue), 10 minutes (yellow), 25 minutes (violet) and 50 minutes (red). MS and MS/MS spectra for each peak are also shown. All ions are observed as $[M-H]^-$. As reaction time is increasing substrate peak (peak 1) is decreasing in intensity, while product peaks are increasing and decreasing, meaning that the substrate is being consumed and the products formed. Major losses during fragmentation are indicated on the MS/MS spectra when relevant. Product peaks are not always characterized by one single mass, but two or more different compound elute at the same time. Whether it is a result of co-elution of two or more different product or chemical degradation has not be investigated. OH-dilignol (peak 1) is identified with the *m/z* 319.05 and a retention time (RT) of 8.3 min. The product profile is simple, only two isomers of the OH-dilignol dimer *m/z* 637.14 are formed (peak 2 and 3) already after 10 minutes of reaction. A sodium formate adduct (+ 68) of the dimer *m/z* 705.12 is present in peak 3. After 50 minutes a compound with *m/z* 455.10 (peak A) appears, showing a mass of 136 higher than OH dilignol (*m/z* 319.05) indicating a double sodium formate adduct of the substrate. Peak * RT 11.3 min, containing *m/z* 293.13 and *m/z* 361.13, where the latter is a sodium formate adduct of *m/z* 293.13, was also found by Petrucci *et al.*[1]. It is, most likely, an impurity of LC-MS eluent system since it is present also in the blank run. Suggested chemical structure for the major products are shown in Supplementary Figure S15.

	possible products from laccase oxidation of sinapic acid
401	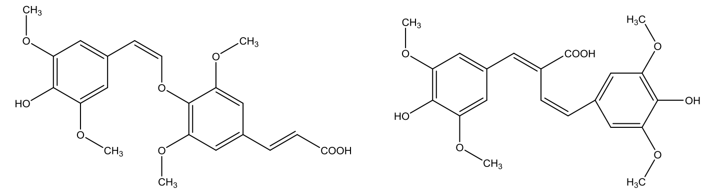
446	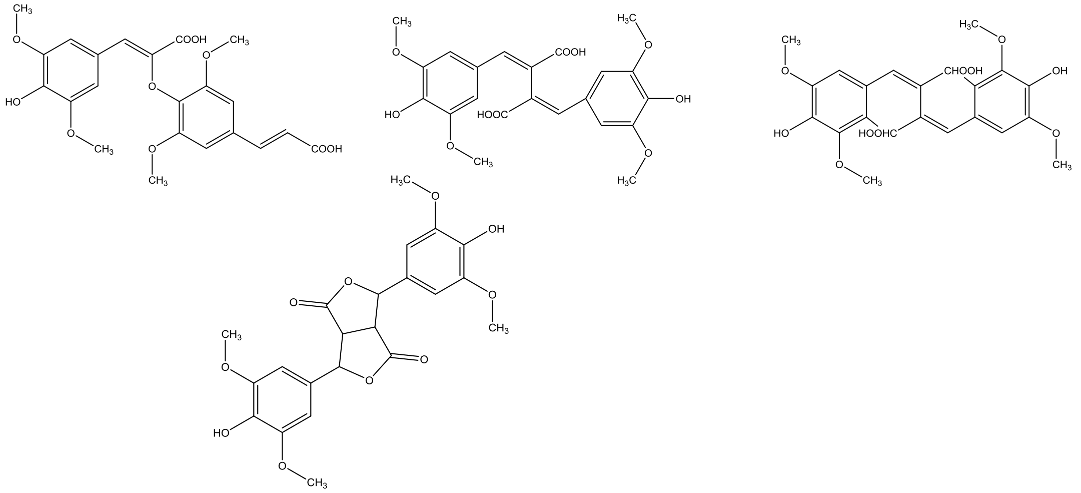

Figure S12. Suggested product structures after laccase oxidation of sinapic acid[2]. Sinapic acid dimer MW 446 g/mol and decarboxylated dimer MW 402 g/mol are the oxidation products fitting the masses found in the reaction evolution profile: Supplementary Figure S2 and S4 peak 3 and 4 and peak 2 and 3, respectively. It has to be noted that these are suggested structures on the basis on what is already known from previous studies[2,3].

possible products from laccase oxidation of ferulic acid	
298	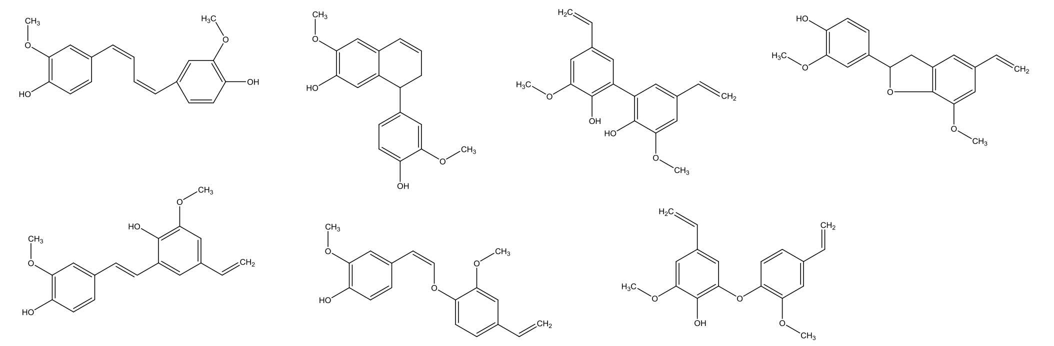
342	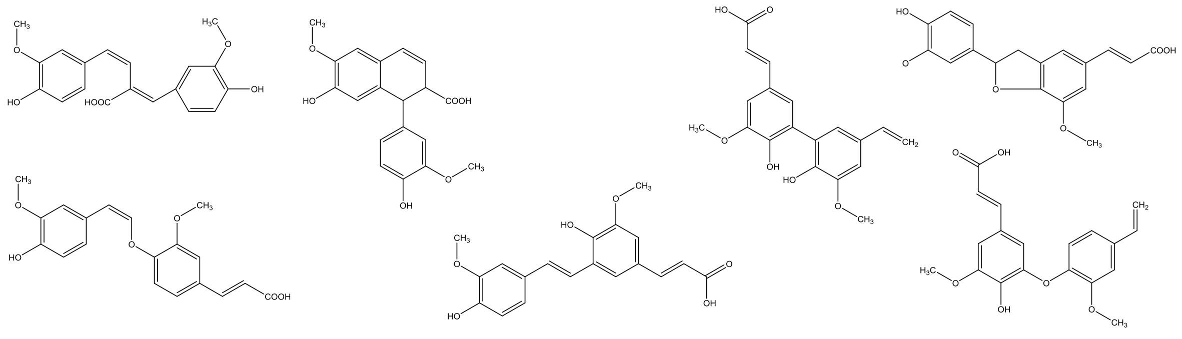
386	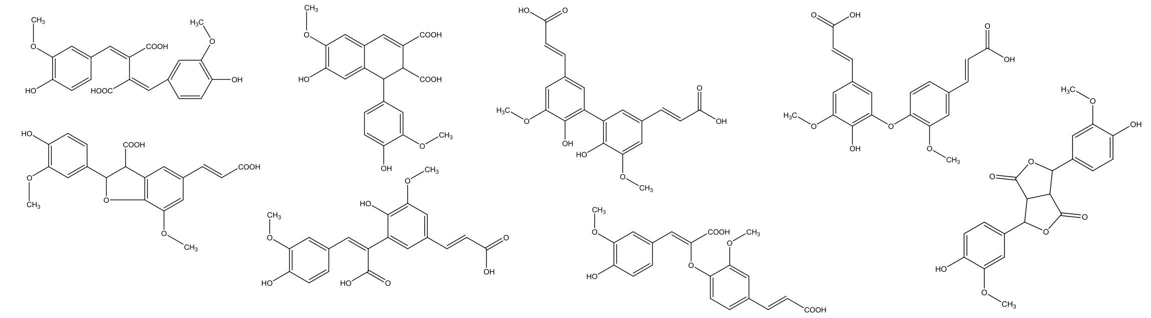

Figure S13. Suggested product structures after laccase oxidation of ferulic acid. Ferulic acid dimer MW 386 g/mol, decarboxylated dimer MW 342 g/mol and doubble decarboxylated dimer MW 298 g/mol are the oxidation products fitting the masses found in the reaction evolution profile: Supplementary Figure S6 and S7 peak 3, 4 and 6 and peak 2, 3 and 4 and peak 4 and 6, respectively. It has to be noted that these are suggested structures on the basis on what is already known from previous studies[3–5].

possible products from laccase oxidation of p-coumaric acid	
282	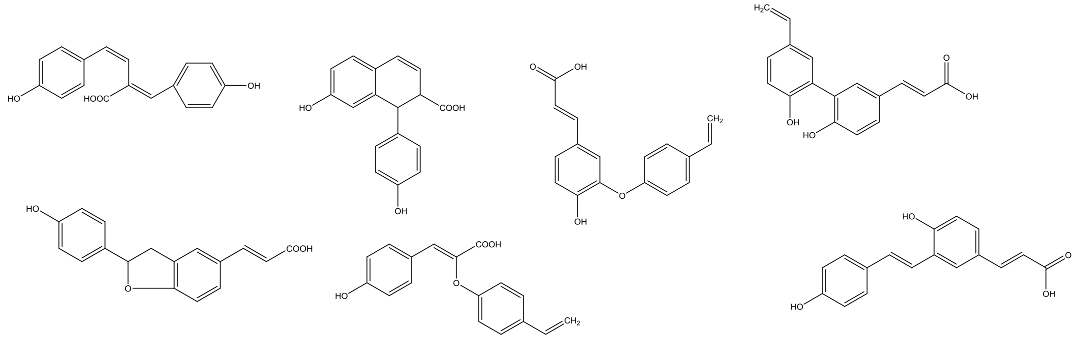
326	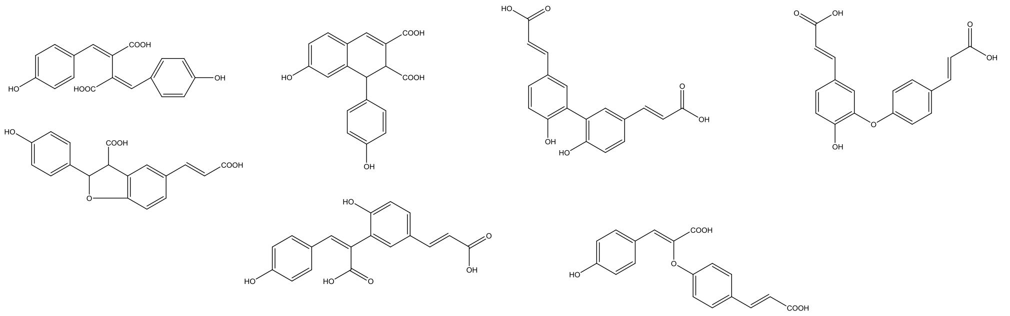
400	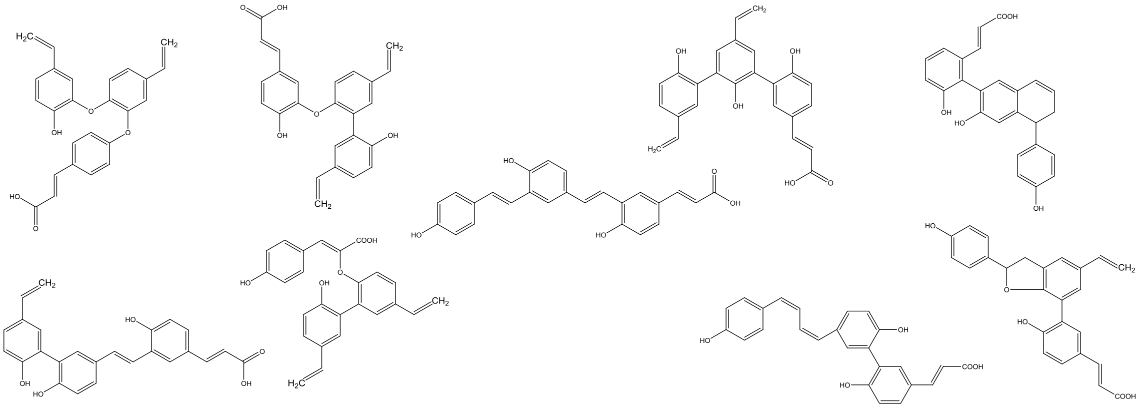
444	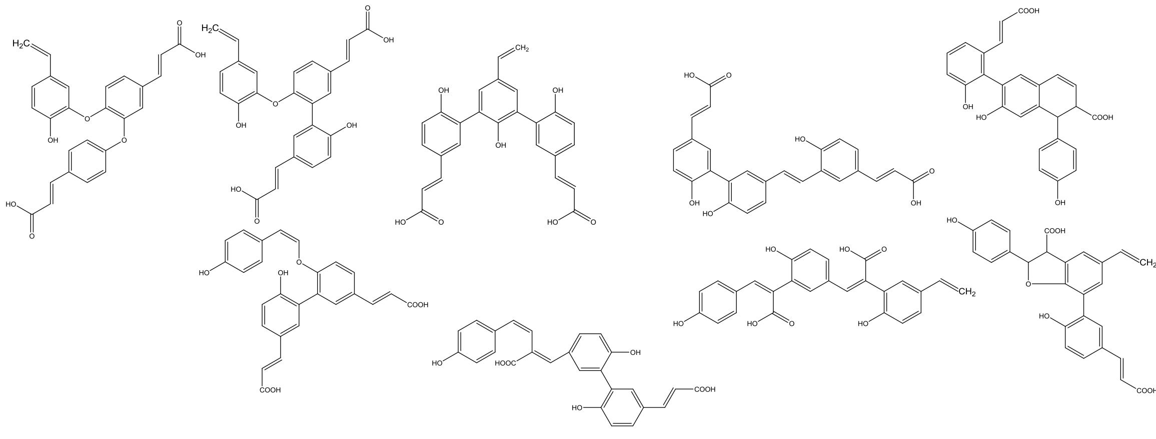

488	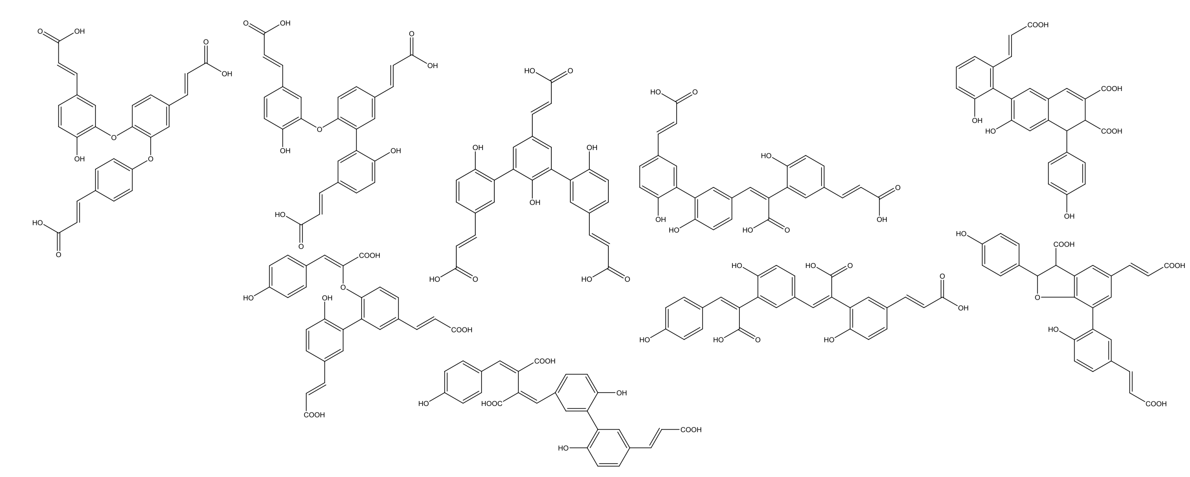
606	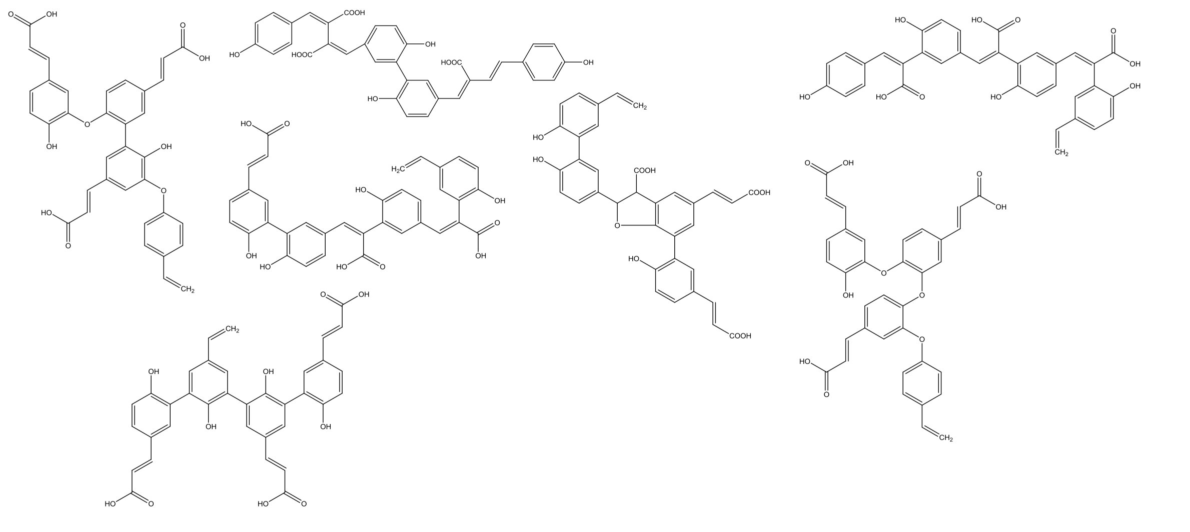

Figure S14. Suggested products structures after laccase oxidation of *p*-coumaric acid. *p*-coumaric acid dimer MW 326 g/mol, decarboxylated dimer MW 282 g/mol, *p*-coumaric acid trimer MW 488 g/mol , decarboxylated trimer MW 444 g/mol, doubble decarboxylated trimer MW 400 g/mol and decarboxylated tetramer 606 g/mol are the oxidation products fitting the masses found in the reaction evolution profile: Supplementary Figure S8 and S9. It has to be noted that these are suggested structures on the basis on what is already known from previous study[3].

possible products from laccase oxidation of OH-dilignol	
638	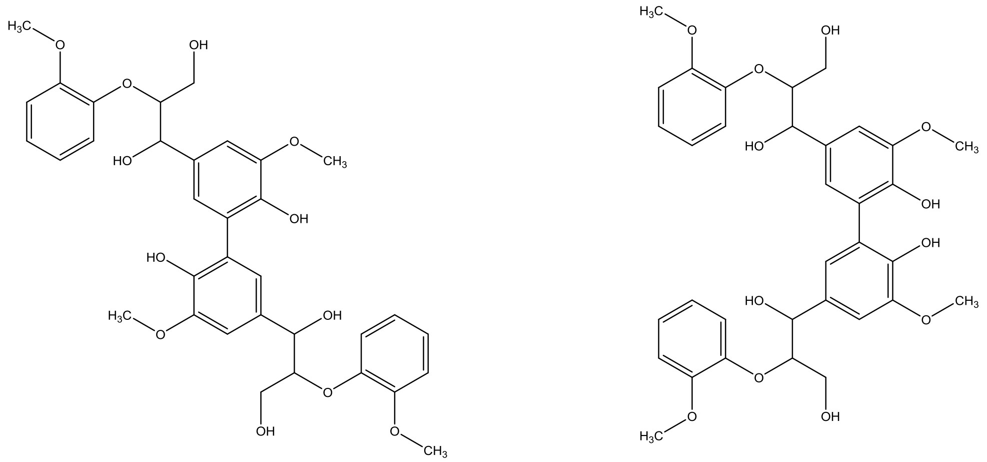

Figure S15. Suggested product structures after laccase oxidation of OH-dilignol. OH-dilignol dimer MW 638 g/mol is the oxidation product fitting the masses found in the reaction evolution profile: Supplementary Figure S10 and S11. Due to the poor conjugated structure, radical propagation outside the aromatic phenolic ring is not possible allowing only formation of a dimer in the para position.

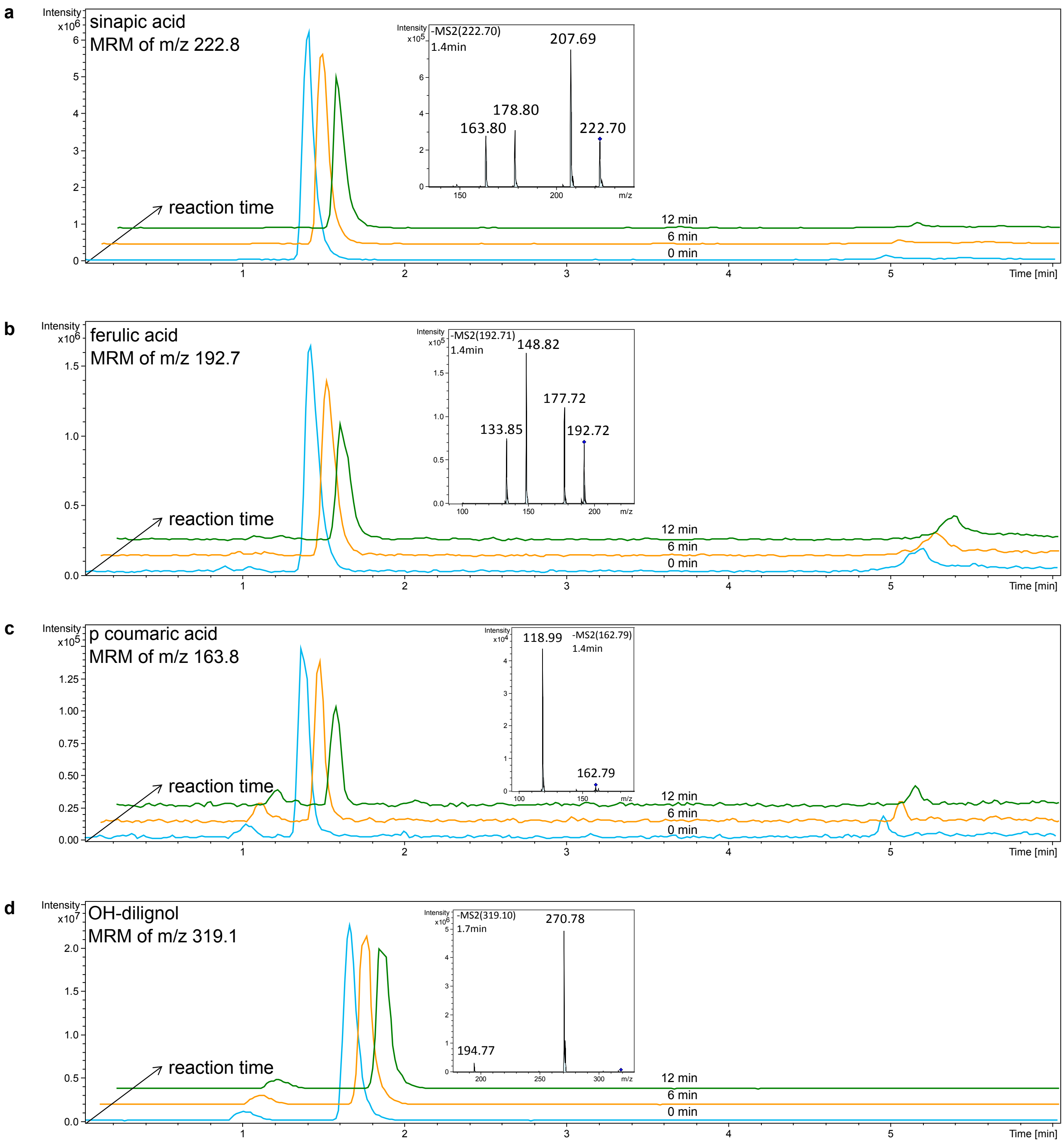

Figure S16. Laccase oxidation evolution profiles for hydroxycinnamic acid and OH-dilignol compound using Multiple Reaction Monitoring (MRM) analysis for substrate quantification: oxidation of sinapic acid (a); oxidation of ferulic acid (b); oxidation of *p*-coumaric acid (c) and oxidation of OH-dilignol (d). Chromatograms show the MRM profile of the oxidation of the different substrates at different reaction time: 0 minutes (light blue), 6 minutes (yellow) and 12 minutes (green). Please note that the intensity scale may differ between chromatograms and is adjusted to give optimal display of figures. Insets show MS/MS of the peak, all the masses found in the MS/MS in addition to the parent mass were used in the quantification methods. All ions are observed as $[M\text{-}H]^{-1}$.

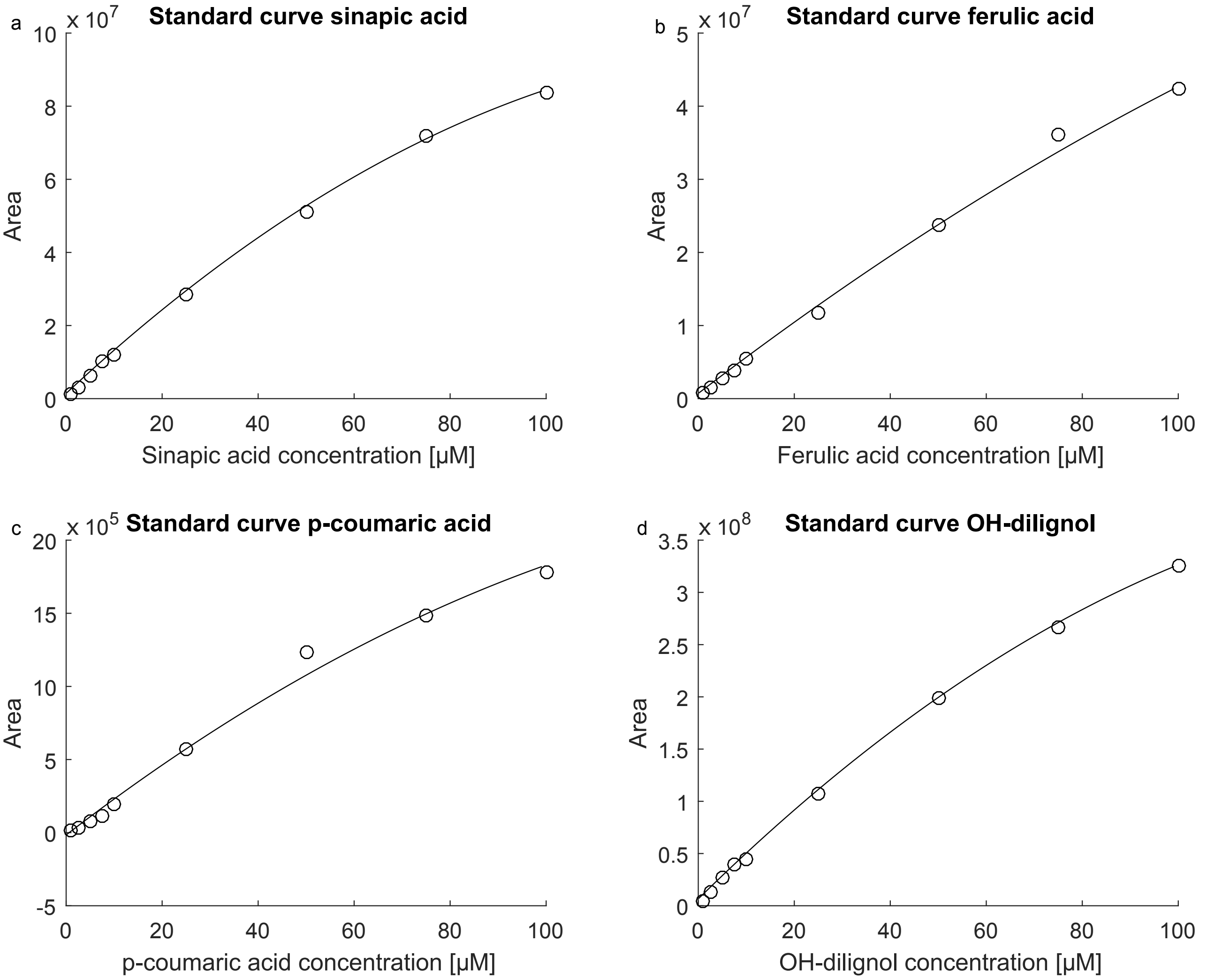

Figure S17. Standard curves for quantification of kinetic: sinapic acid (a), ferulic acid (b), *p*-coumaric acid (c) and OH-dilignol (d). The area is the target area determined as sum of the the different masses reported in Supplementary Figure S16. Nine different level of standards were used: 1, 2.5, 5, 7.5, 10, 25, 50, 75 and 100 μM. Data are fitted with a quadratic curve. Y-axis is optimized in order to have a optimal view of the curves.

References

1. A spectroelectrochemical and chemical study on oxidation of hydroxycinnamic acids in aprotic medium. *Electrochimica Acta* **52**, 2461–2470 (2007).

2. Lacki, K. & Duvnjak, Z. Transformation of 3,5-dimethoxy,4-hydroxy cinnamic acid by polyphenol oxidase from the fungusTrametes versicolor: Product elucidation studies. *Biotechnol. Bioeng.* **57**, 694–703 (1998).

3. Vismeh, R. *et al.* Profiling of diferulates (plant cell wall cross-linkers) using ultrahigh-performance liquid chromatography-tandem mass spectrometry. *The Analyst* **138**, 6683 (2013).

4. Ralph, J. *et al.* Lignins: Natural polymers from oxidative coupling of 4-hydroxyphenyl- propanoids. *Phytochem. Rev.* **3**, 29–60 (2004).

5. Carunchio, F., Crescenzi, C., Girelli, A. M., Messina, A. & Tarola, A. M. Oxidation of ferulic acid by laccase: identification of the products and inhibitory effects of some dipeptides. *Talanta* **55**, 189–200 (2001).
